# Supplementary material for: How stra(i)nge are your controls? A comparative analysis of metabolic phenotypes in commonly used C57BL/6 substrains
Source: PLoS One. 2023 Aug 2;18(8):e0289472. doi: 10.1371/journal.pone.0289472 (PMC10395817; doi:10.1371/journal.pone.0289472)
Supplement: S1 File — (PDF) [file pone.0289472.s005.pdf]

# Raw blot and total protein stain (Ponceau) images

# Female substrain blots (Fig. 3A)

# p-AKT for Fig. 3A

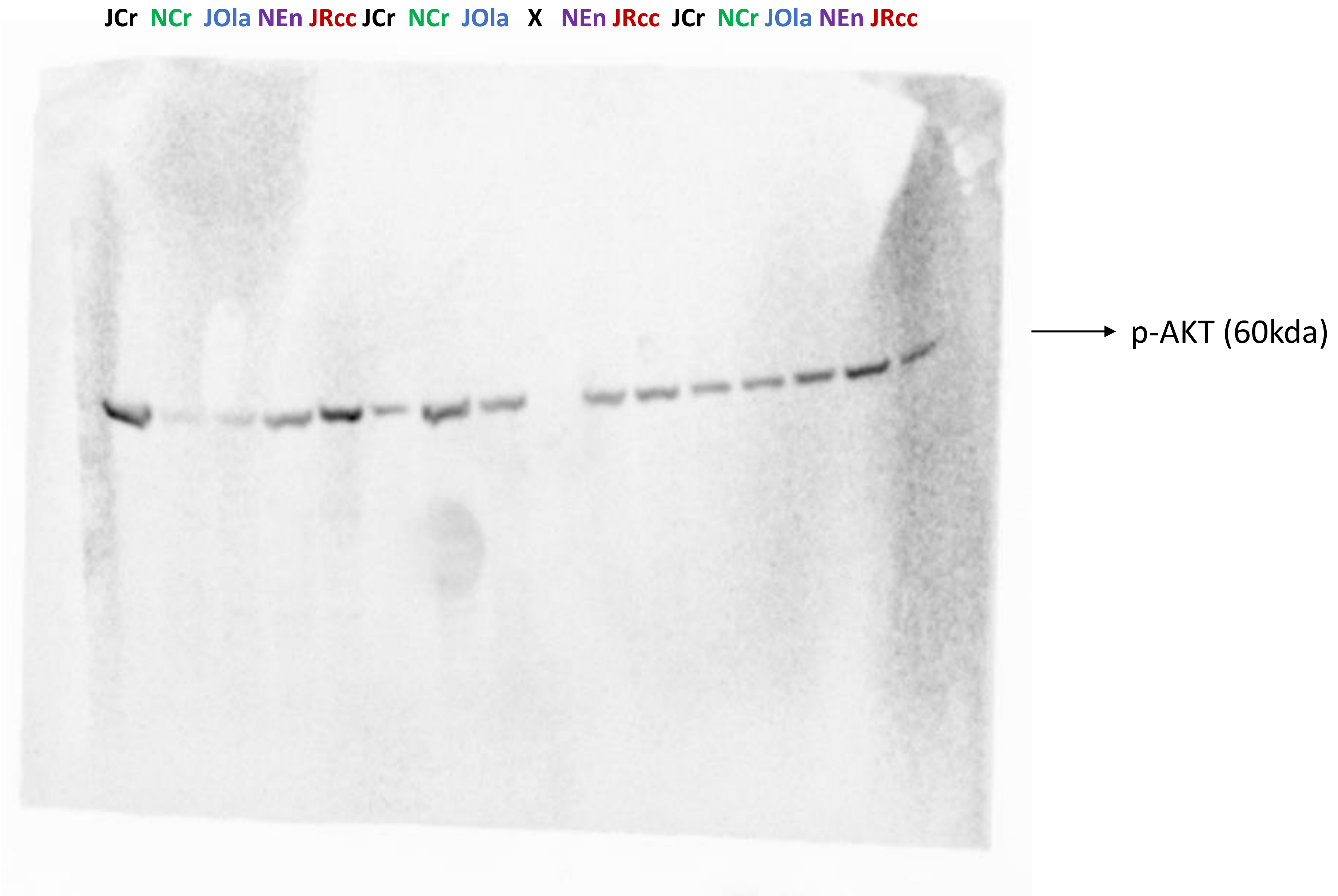


**Annotated Groups:**

JCr =6JCRL (Charles River)

NCr =6NCRL

JOla=6JOlaEnv (Envigo)

NEn =6NEnv

JRcc=6JRccEnv

**Method used:**

visualised using Enhanced chemiluminescence substrate on iBright™ FL1000 Imaging System camera (Invitrogen™, Fisher Scientific) at 16-bit

# t-AKT for Fig. 3A

JCr NCr JOla NEn JRcc JCr NCr JOla X NEn JRcc JCr NCr JOla NEn JRcc

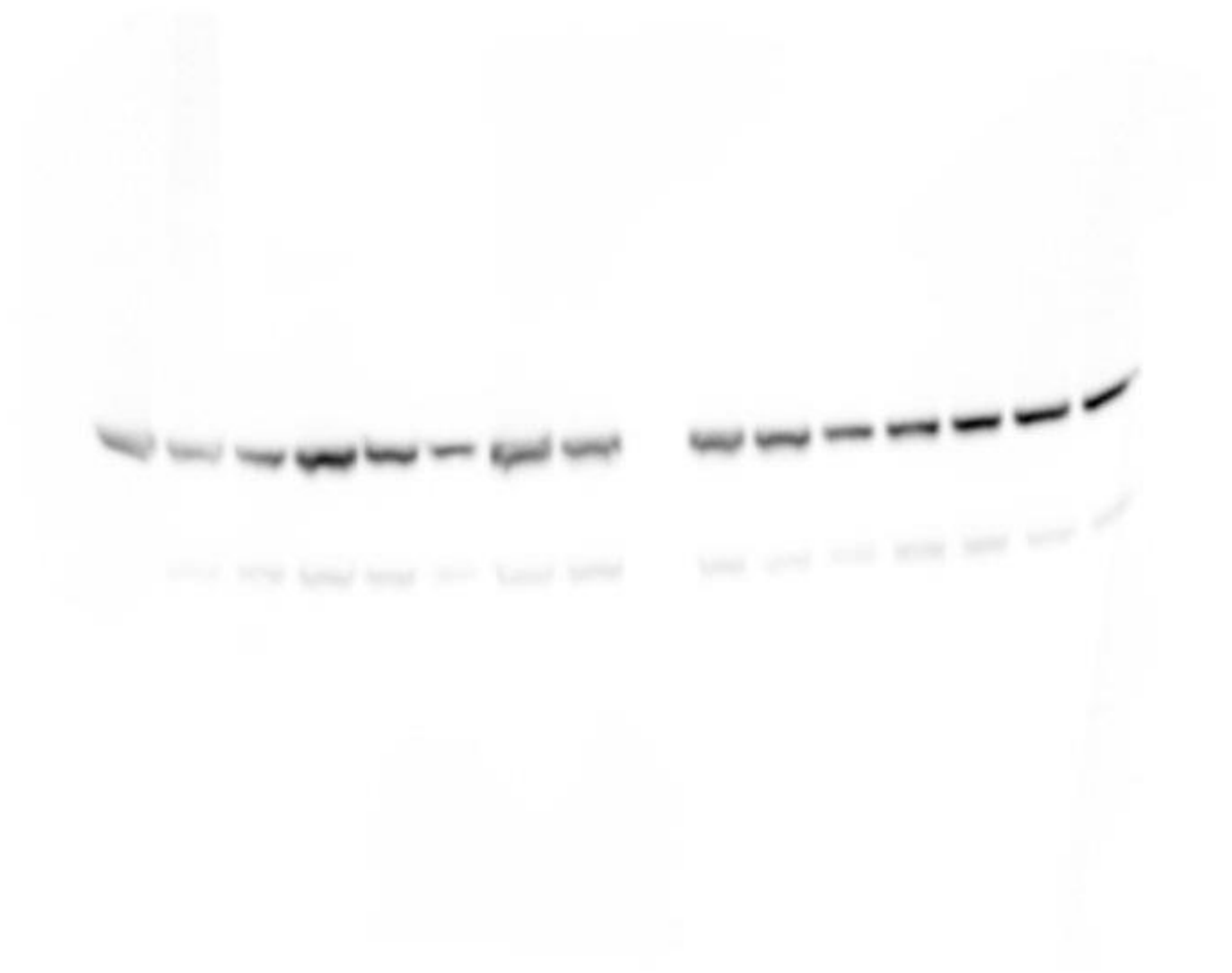

t-AKT
60kDa

**Annotated Groups:**
JCr =6JCRL (Charles River)
NCr =6NCRL
JOla=6JOlaEnv (Envigo)
NEn =6NEnv
JRcc=6JRccEnv

**Method used:**
visualised using Enhanced chemiluminescencse ubstrate on iBright™ FL1000 Imaging System camera (Invitrogen™, Fisher Scientific) at 16-bit

# Ponceau for normalisation of  p-AKT and t-AKT in Fig. 3A

SB JCr NCr JOla NEn JRcc JCr NCr JOla X NEn JRcc JCr NCr JOla NEn JRcc

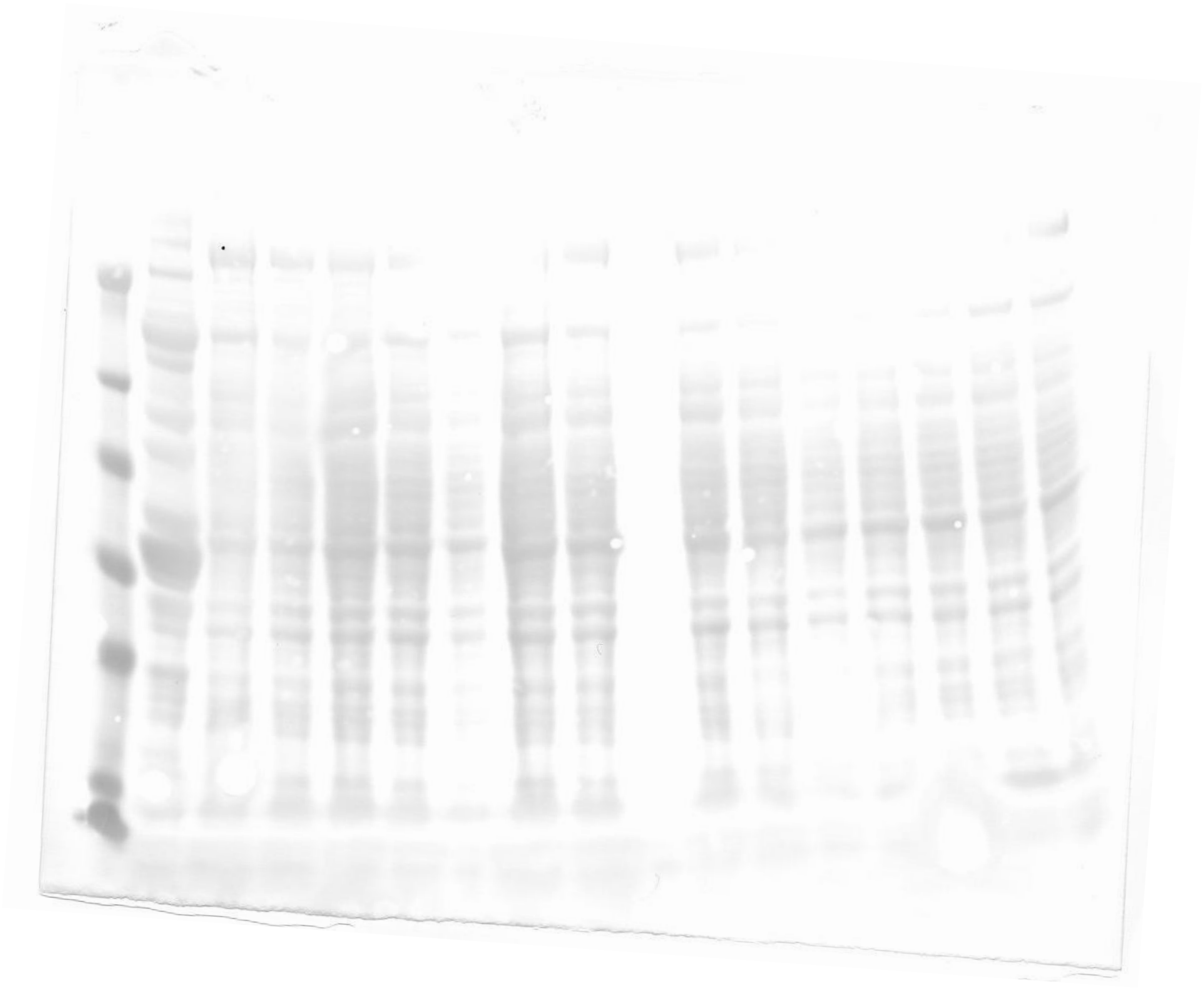

**Annotated Groups:**
SB=Seeblue
JCr =6JCRL (Charles River)
NCr =6NCRL
JOla=6JOlaEnv (Envigo)
NEn =6NEnv
JRcc=6JRccEnv

**Method used:**
visualised using Enhanced chemiluminescence substrate on iBright™ FL1000 Imaging System camera (Invitrogen™, Fisher Scientific) at 16-bit

# p-rpS6 for Fig. 3A

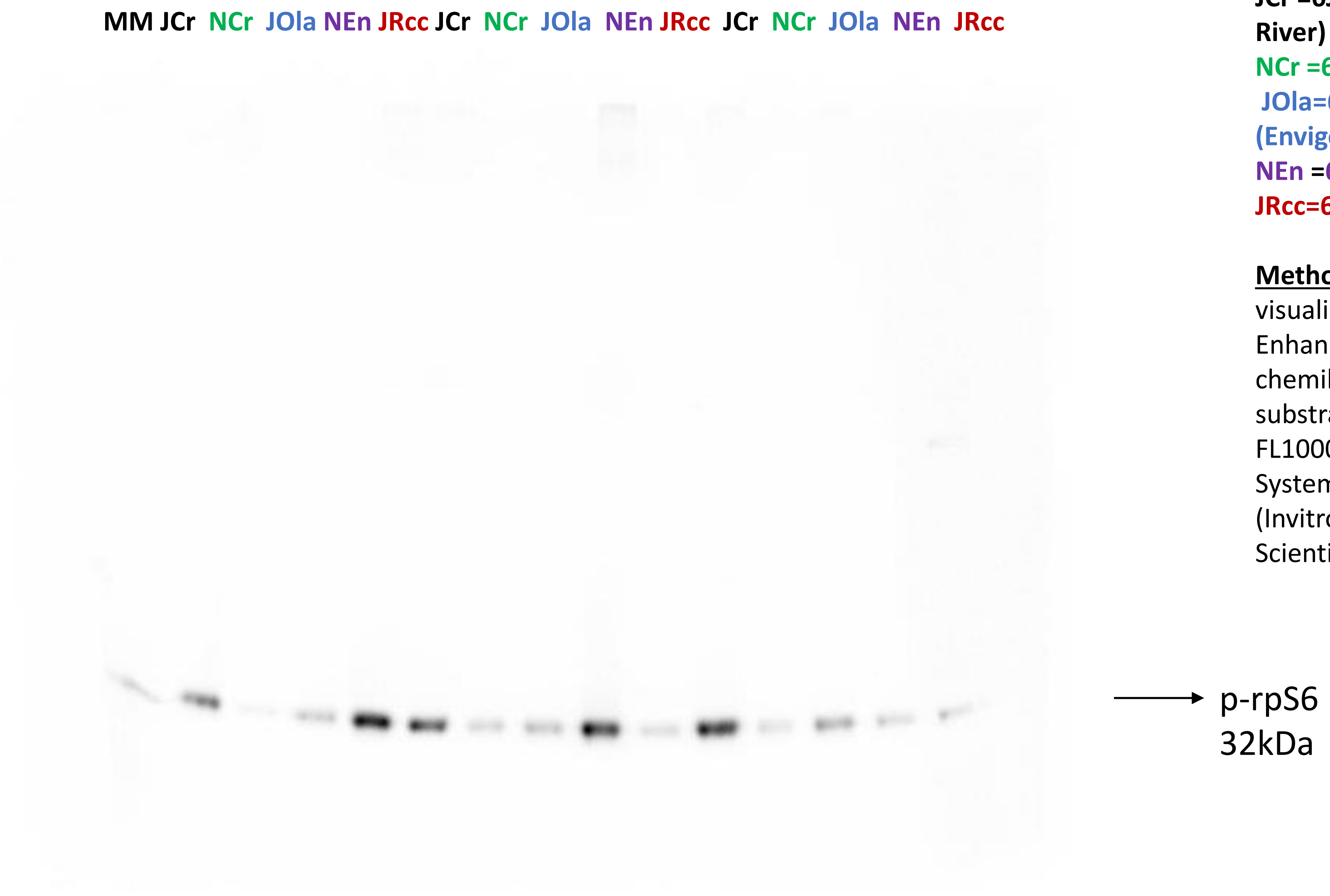


**Annotated Groups:**
**MM=Magic Mark XP**
**JCr =6JCRL (Charles River)**
**NCr =6NCRL**
**JOla=6JOlaEnv (Envigo)**
**NEn =6NEnv**
**JRcc=6JRccEnv**

**Method used:**
visualised using Enhanced chemiluminescence substrate on iBright™ FL1000 Imaging System camera (Invitrogen™, Fisher Scientific) at 16-bit

# t-rpS6 for Fig. 3A

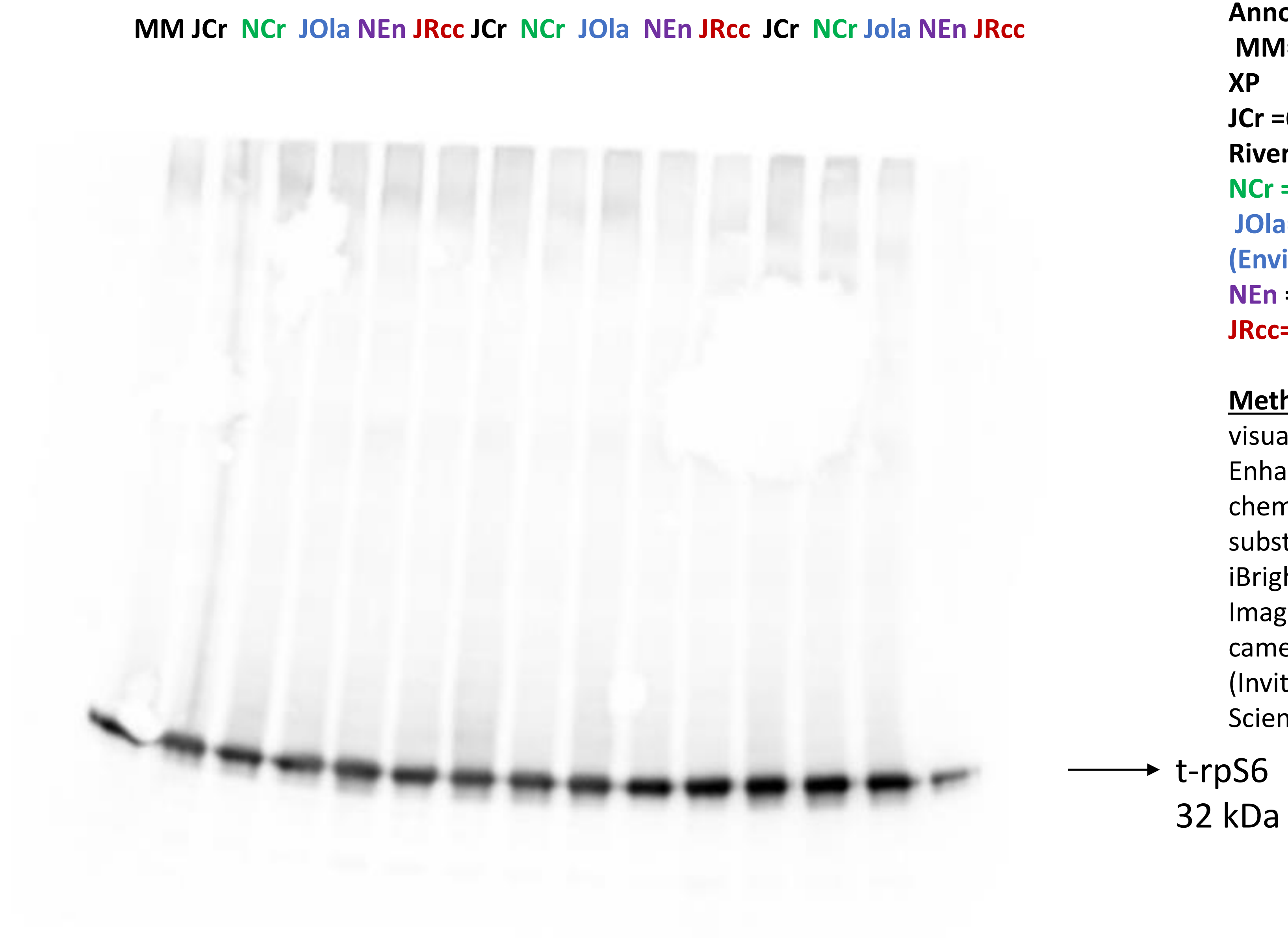


**Annotated Groups:**
**MM=Magic Mark XP**
**JCr =6JCRL (Charles River)**
**NCr =6NCRL**
**JOla=6JOlaEnv (Envigo)**
**NEn =6NEnv**
**JRcc=6JRccEnv**

**Method used:**
visualised using Enhanced chemiluminescencs substrate on iBright™ FL1000 Imaging System camera (Invitrogen™, Fisher Scientific) at 16-bit

# p-GSK3β for Fig. 3A

MM JCr NCr JOla NEn JRcc JCr NCr JOla NEn JRcc JCr NCr JOla NEn JRcc

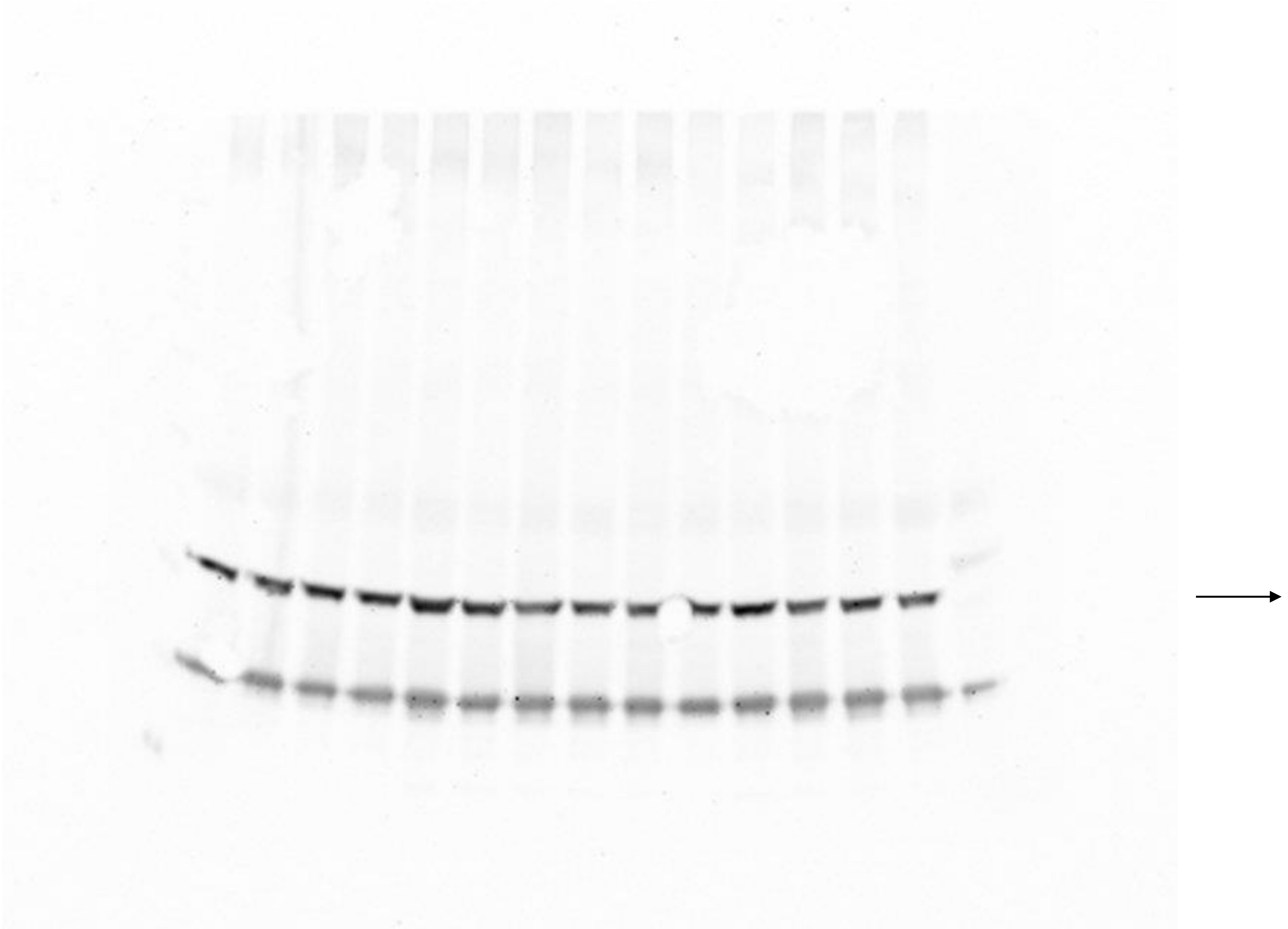


**Annotated Groups:**
**MM=Magic Mark XP**
**JCr =6JCRL (Charles River)**
**NCr =6NCRL**
**JOla=6JOlaEnv (Envigo)**
**NEn =6NEnv**
**JRcc=6JRccEnv**

**Method used:**
visualised using Enhanced chemiluminescencssubstrate on iBright™ FL1000 Imaging System camera (Invitrogen™, Fisher Scientific) at 16-bit

# t-GSK3β for Fig. 3A

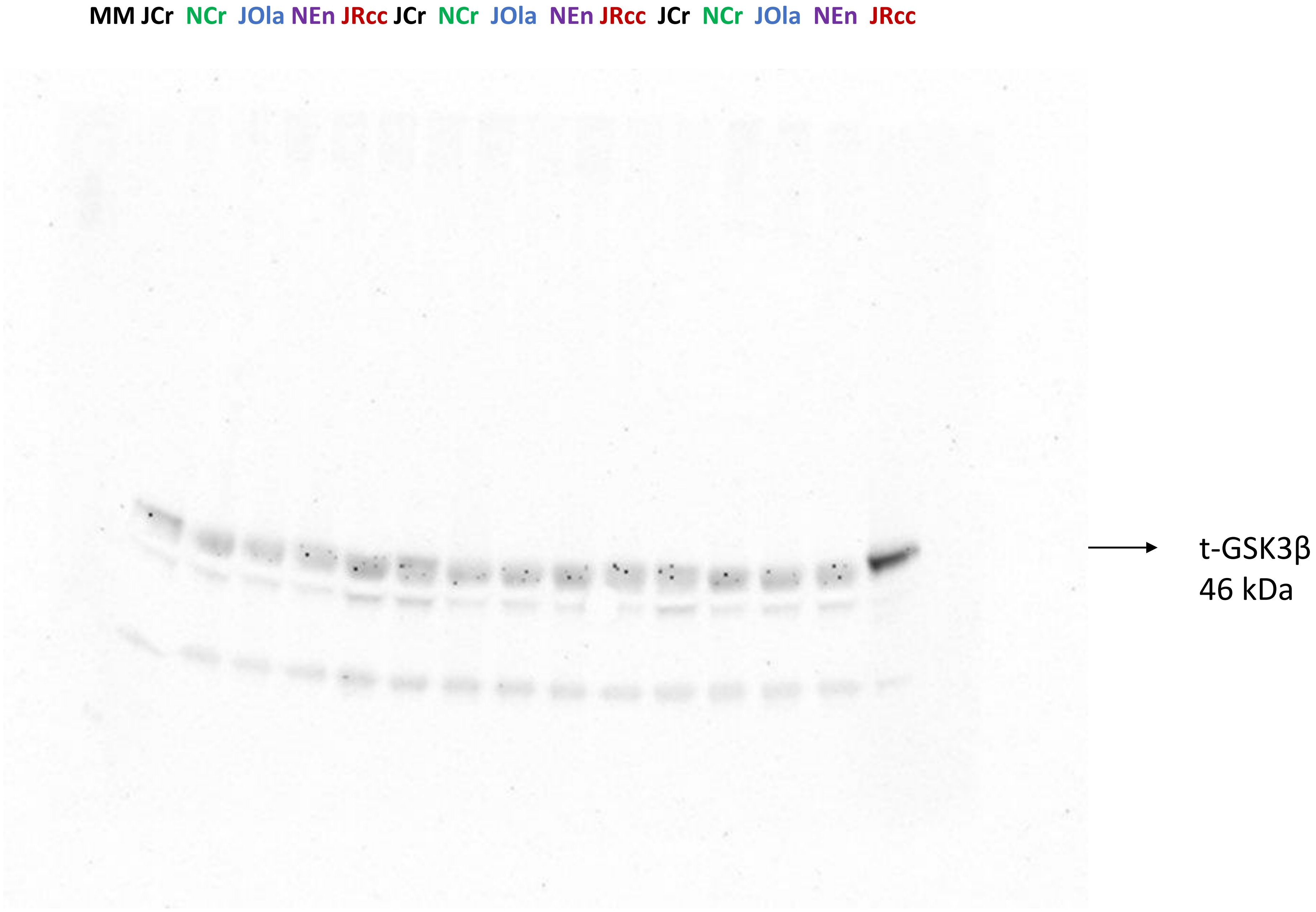


**Annotated Groups:**
MM=Magic Mark XP
JCr =6JCRL (Charles River)
NCr =6NCRL
JOla=6JOlaEnv (Envigo)
NEn =6NEnv
JRcc=6JRccEnv

**<u>Method used:</u>**
visualised using Enhanced chemiluminescencssubstrate on iBright™ FL1000 Imaging System camera (Invitrogen™, Fisher Scientific) at 16-bit

# Ponceau for normalisation of AKT and GSK3B (both p- and t-)in Fig. 3A

**JCr NCr JOla NEn JRcc JCr NCr JOla NEn JRcc JCr NCr JOla NEn JRcc** **SB**

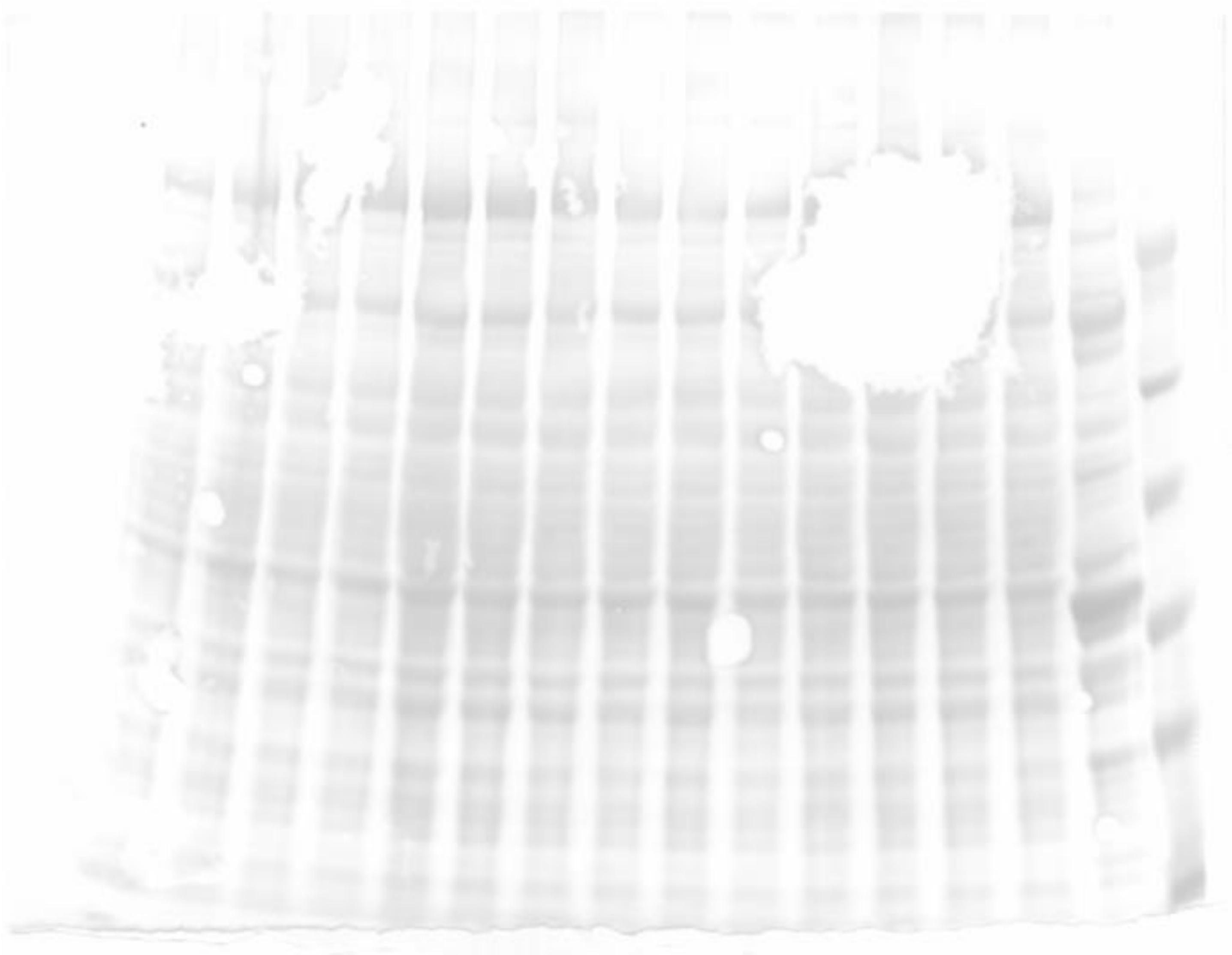

**Annotated Groups:**
**JCr =6JCRL (Charles River)**
**NCr =6NCRL**
**JOla=6JOlaEnv (Envigo)**
**NEn =6NEnv**
**JRcc=6JRccEnv**
**SB = Seeblue**

**Method used:**
visualised using Enhanced chemiluminescence substrate on iBright™ FL1000 Imaging System camera (Invitrogen™, Fisher Scientific) at 16-bit

# Male substrain blots (Fig. 3D)

# p-AKT for Fig. 3D

MM JCr JCr NCr NCr JOla NEnNEn JRcc JRcc JCr NCrJOlaNEnJRcc JRcc

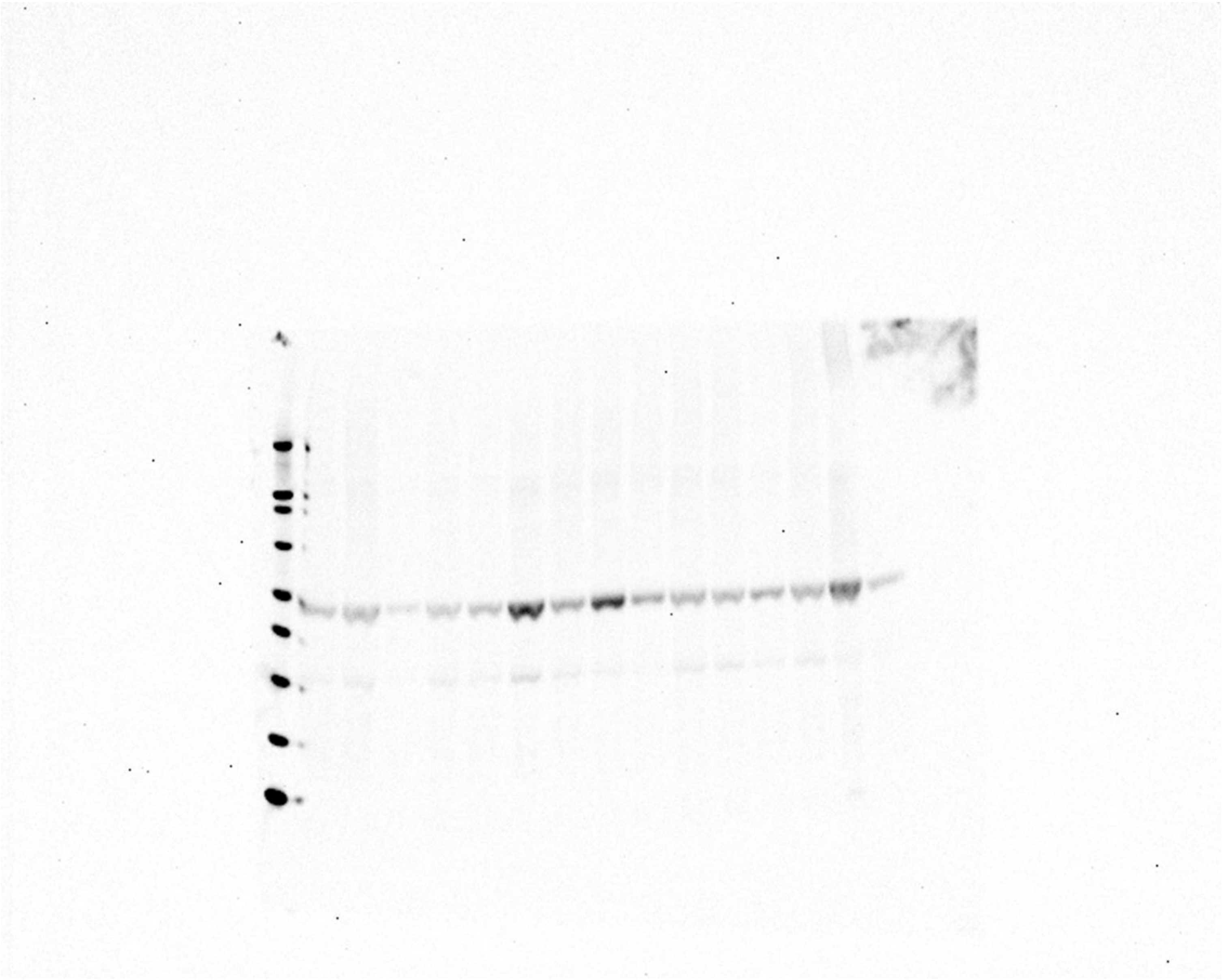

p-AKT
60 kDa

**Annotated Groups:**
**MM=Magic Mark XP**
**JCr =6JCRL (Charles River)**
**NCr =6NCRL**
**JOla=6JOlaEnv (Envigo)**
**NEn =6NEnv**
**JRcc=6JRccEnv**

**Method used:**
visualised using Enhanced chemilumiscence substrate on iBright™ FL1000 Imaging System camera (Invitrogen™, Fisher Scientific) at 16-bit

# t-AKT for Fig. 3D

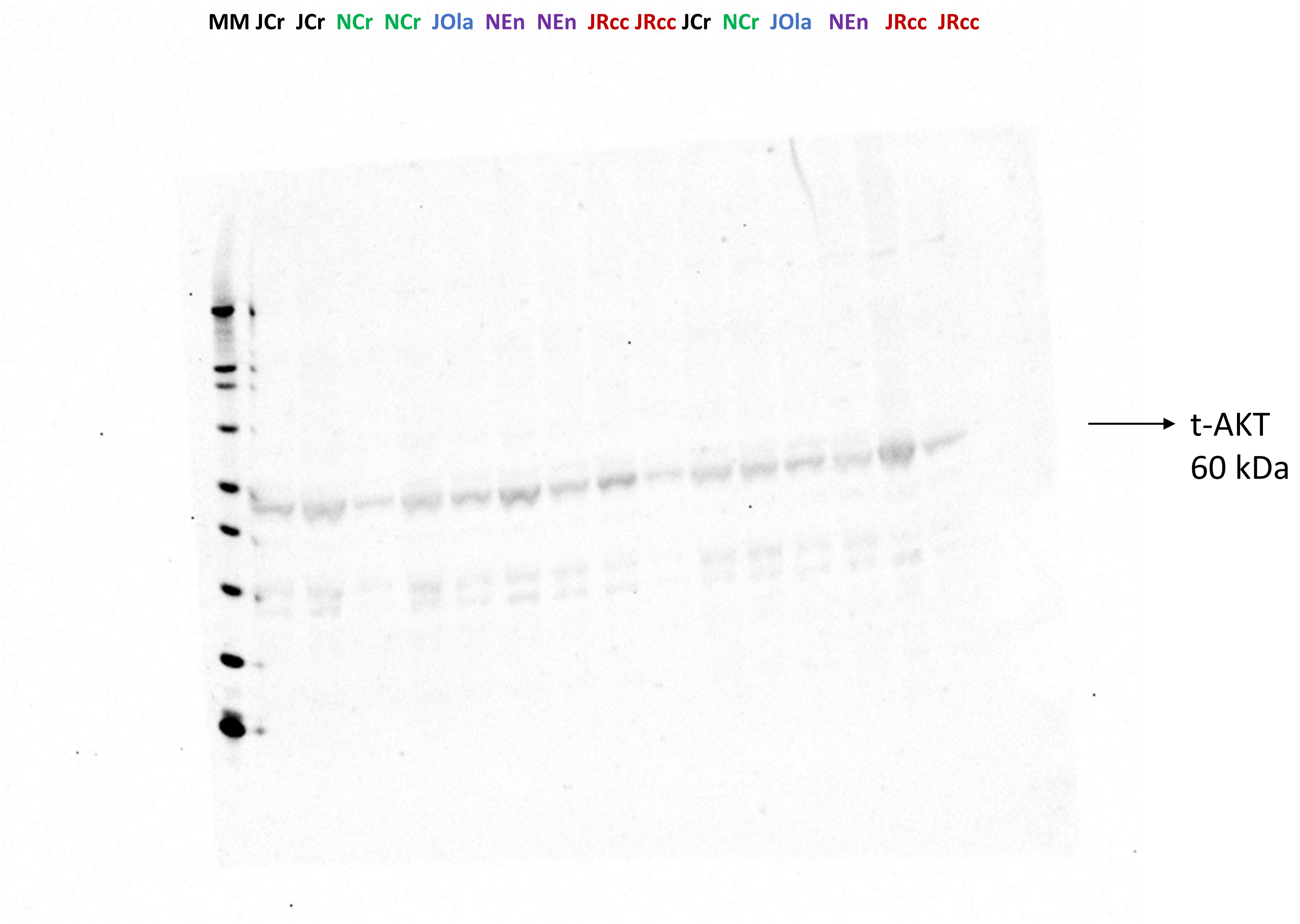


**Annotated Groups:**
MM=Magic Mark XP
JCr =6JCRL (Charles River)
NCr =6NCRL
JOla=6JOlaEnv (Envigo)
NEn =6NEnv
JRcc=6JRccEnv

**Method used:**
visualised using Enhanced chemiluminescence substrate on iBright™ FL1000 Imaging System camera (Invitrogen™, Fisher Scientific) at 16-bit

# Ponceau for normalisation of  p-AKT and t-AKT in Fig. 3D

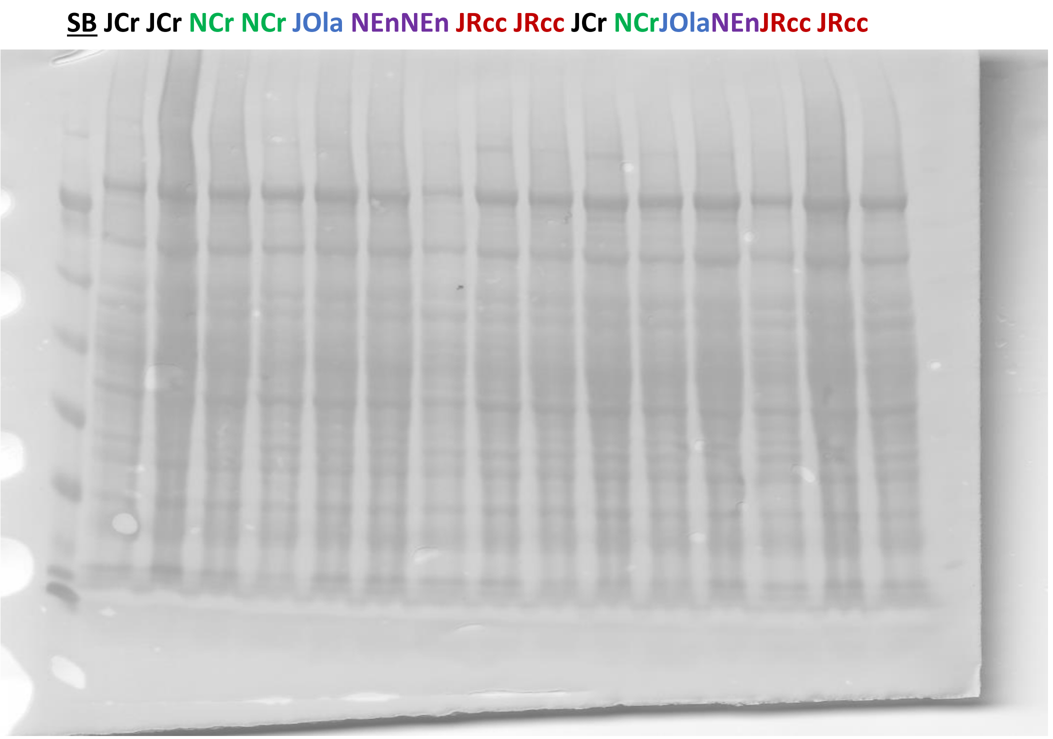


**Annotated Groups:**
**MM=Magic Mark XP**
**JCr =6JCRL (Charles River)**
**NCr =6NCRL**
**JOla=6JOlaEnv (Envigo)**
**NEn =6NEnv**
**JRcc=6JRccEnv**

**Method used:**
visualised using Enhanced chemilumiscence substrate on iBright™ FL1000 Imaging System camera (Invitrogen™, Fisher Scientific) at 16-bit

# p-rpS6 for Fig. 3D

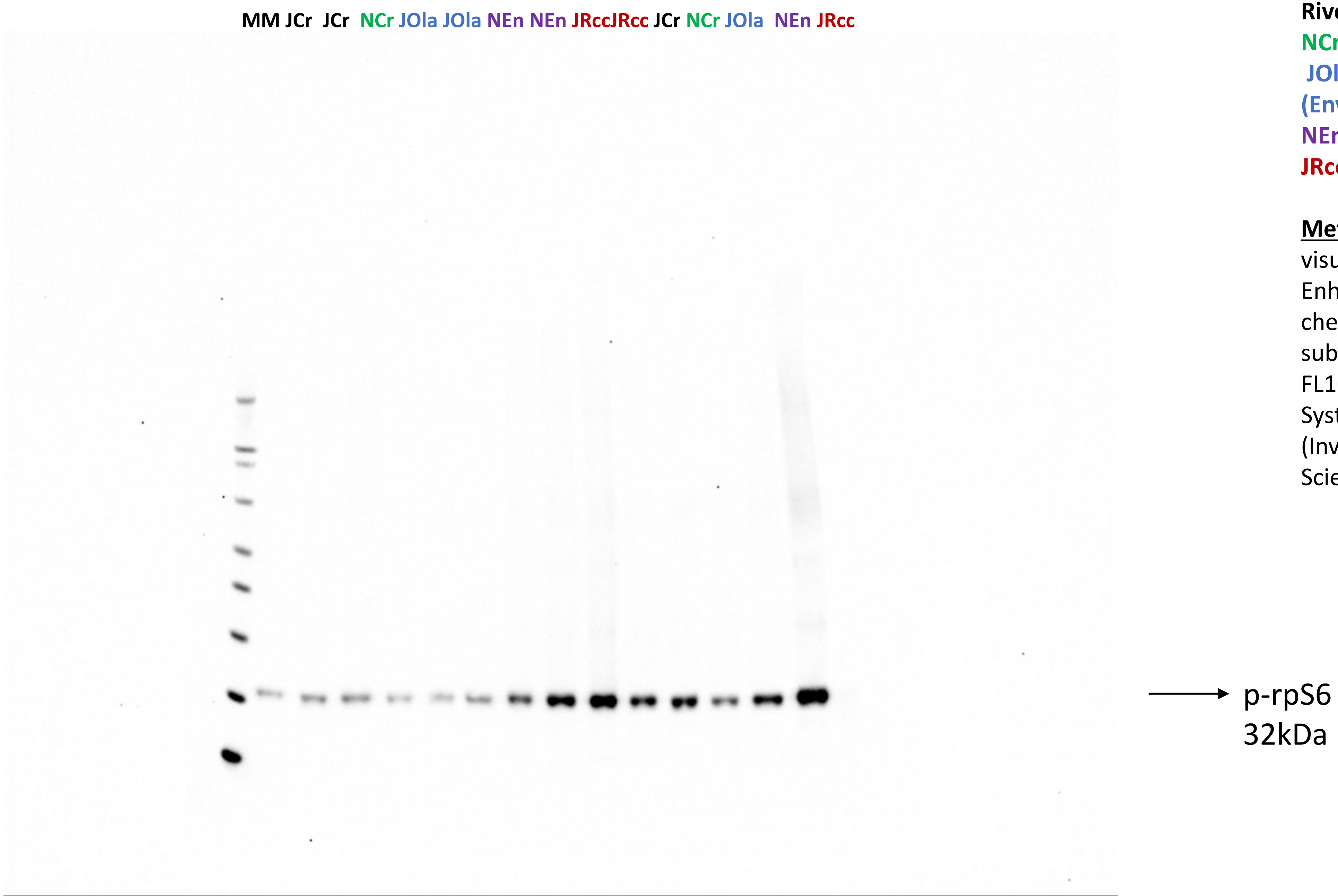


**Annotated Groups:**
**MM=Magic Mark XP**
**JCr =6JCRL (Charles River)**
**NCr =6NCRL**
**JOla=6JOlaEnv (Envigo)**
**NEn =6NEnv**
**JRcc=6JRccEnv**

**Method used:**
visualised using Enhanced chemiluminescence substrate on iBright™ FL1000 Imaging System camera (Invitrogen™, Fisher Scientific) at 16-bit

# t-rpS6 for Fig. 3D

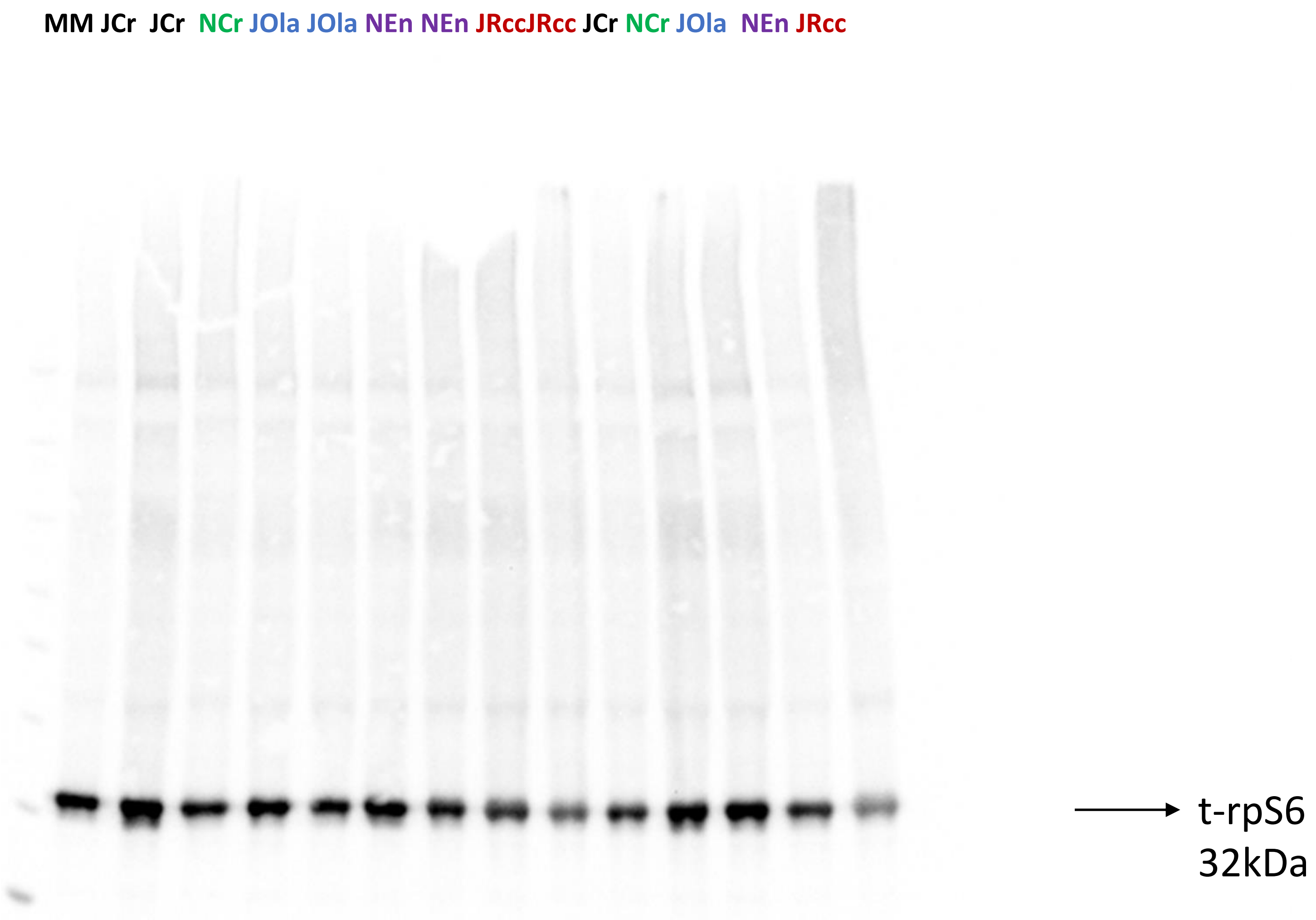


**Annotated Groups:**
**MM=Magic Mark XP**
**JCr =6JCRL (Charles River)**
**NCr =6NCRL**
**JOla=6JOlaEnv (Envigo)**
**NEn =6NEnv**
**JRcc=6JRccEnv**

**Method used:**
visualised using Enhanced chemiluminescence substrate on iBright™ FL1000 Imaging System camera (Invitrogen™, Fisher Scientific) at 16-bit

# Ponceau for normalisation of p-rpS6 and t-rpS6 in Fig. 3D

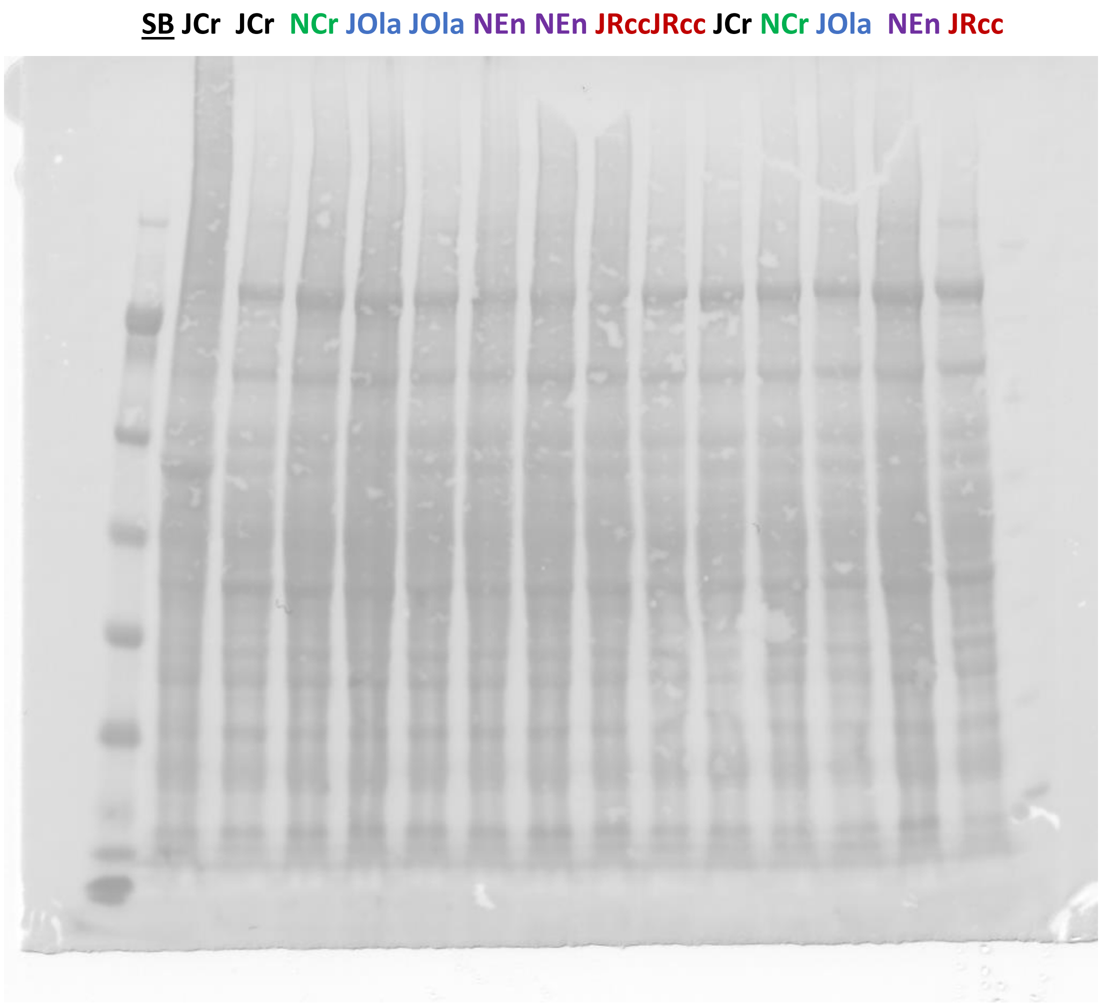


**Annotated Groups:**
**MM=Magic Mark XP**
**SB = Seeblue**
**JCr =6JCRL (Charles River)**
**NCr =6NCRL**
**JOla=6JOlaEnv (Envigo)**
**NEn =6NEnv**
**JRcc=6JRccEnv**

**Method used:**
visualised using Enhanced chemiluminescence substrate on iBright™ FL1000 Imaging System camera (Invitrogen™, Fisher Scientific) at 16-bit

# p-GSK3β for Fig. 3D

Original blot photo from camera

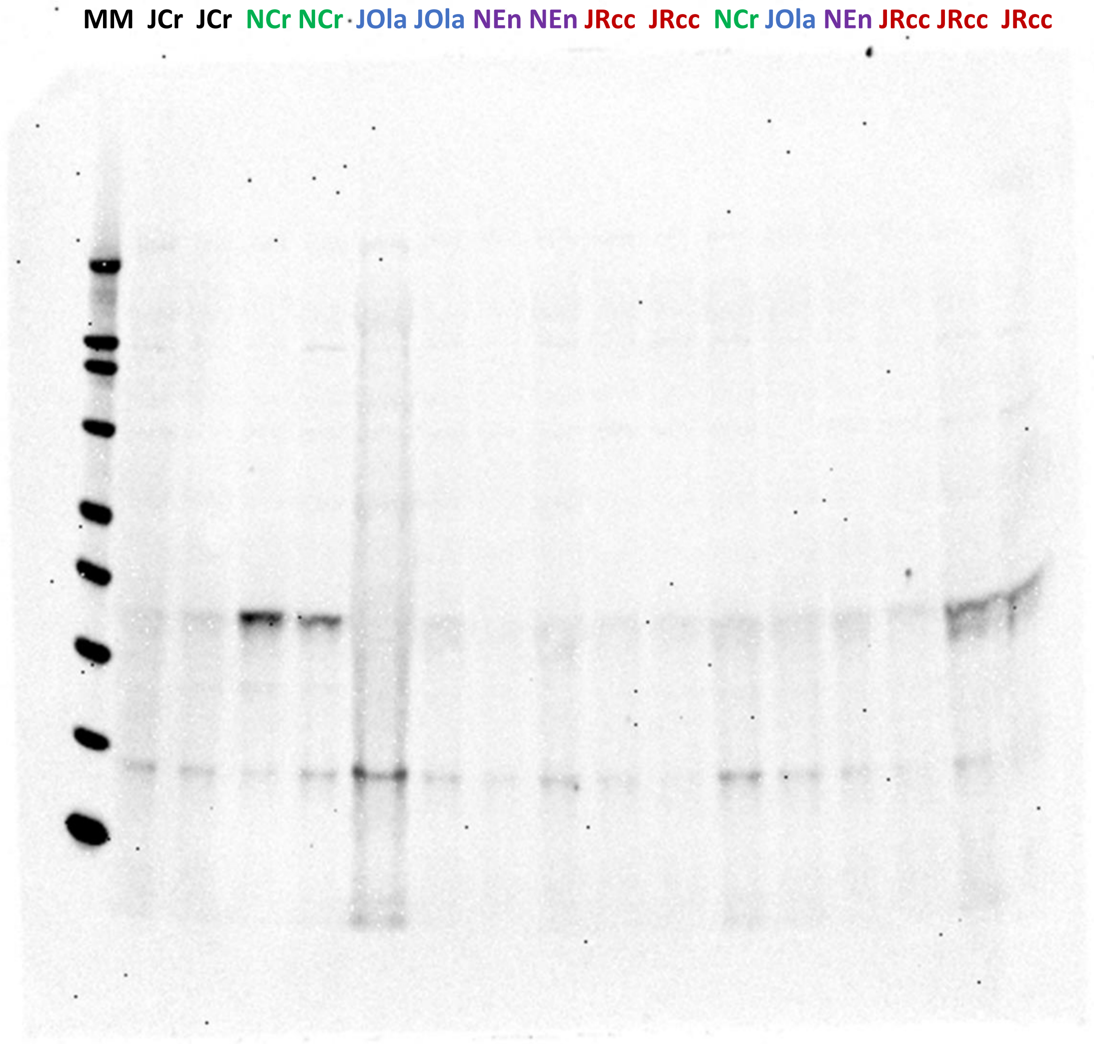


Contrast ramped up on Image J to analyze, matching blot photo in Fig 3D

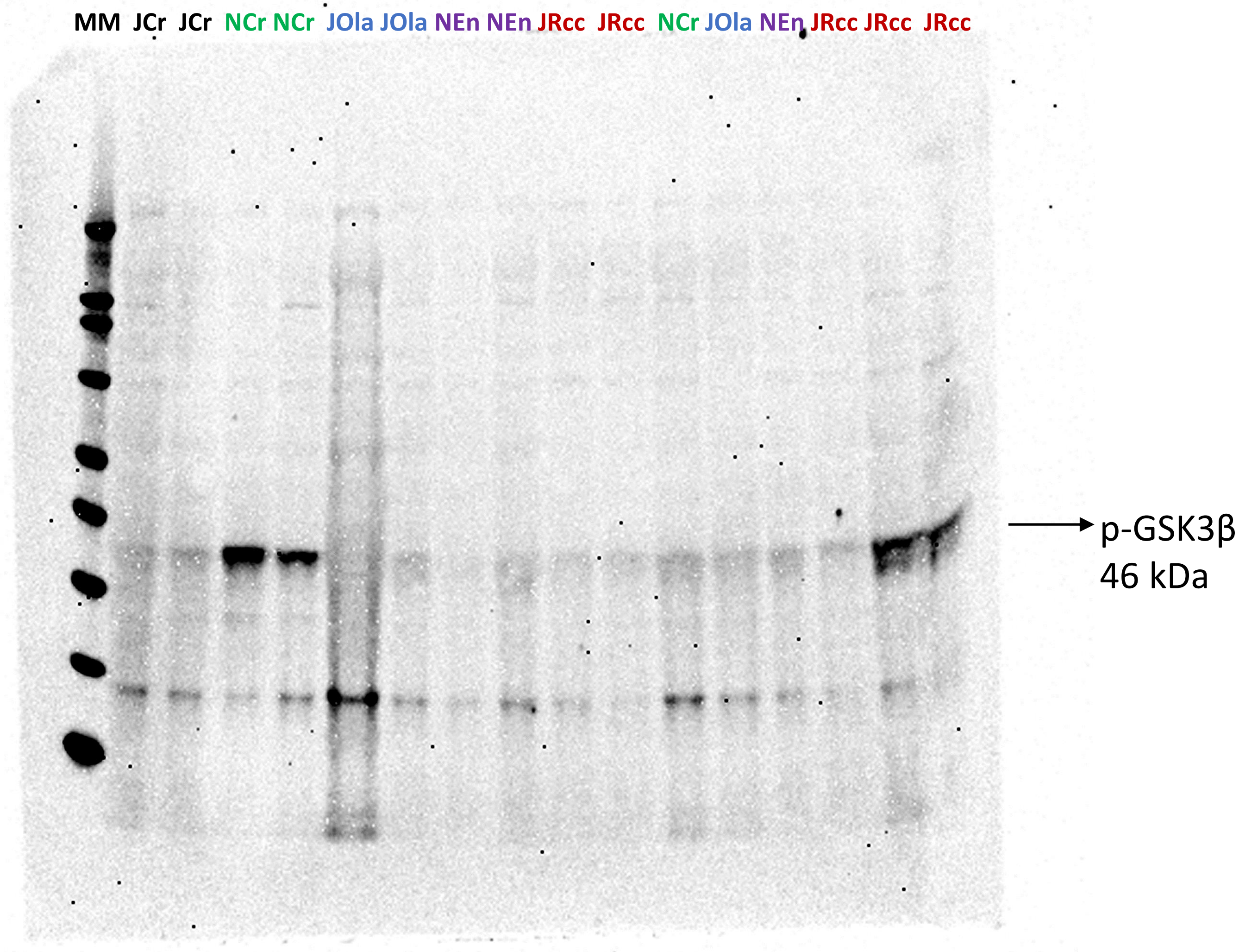


**Annotated Groups:** **MM=Magic Mark XP, JCr =6JCRL (Charles River), NCr =6NCRL, JOla=6JOlaEnv (Envigo), NEn =6NEnv, JRcc=6JRccEnv**

**Method used:** visualised using Enhanced chemiluminescence substrate on iBright™ FL1000 Imaging System camera (Invitrogen™, Fisher Scientific) at 16-bit

# t-GSK3β for Fig. 3D

Original blot photo from camera

MM JCr JCr NCr NCr JOla JOla NEn NEn JRcc JRcc NCr JOla NEn JRcc JRcc JRcc

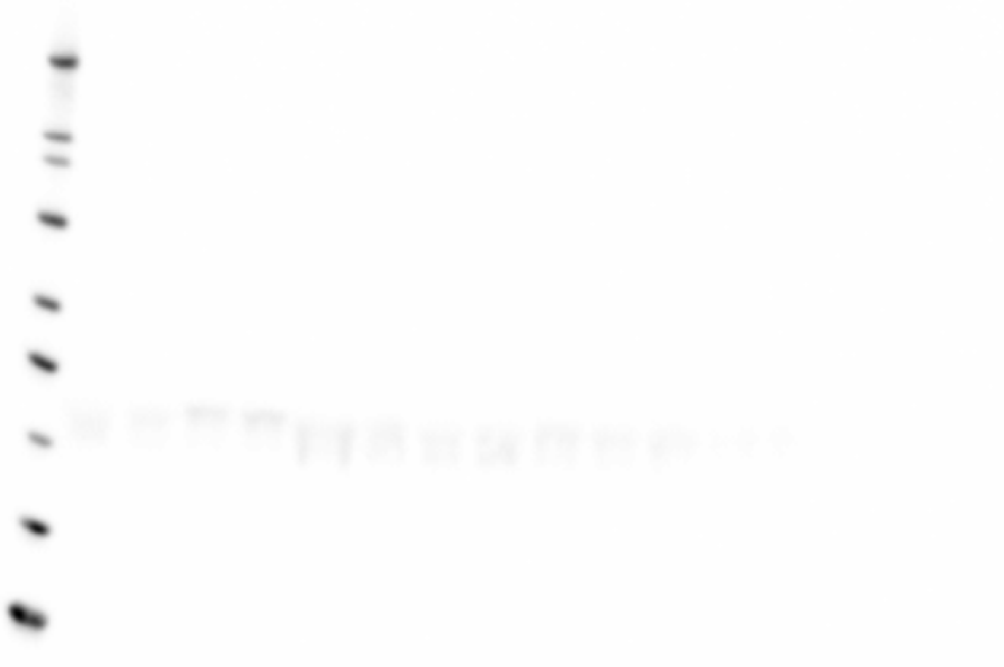

Contrast ramped up on Image J to analyze, matching blot photo in Fig 3D

MM JCr JCr NCr NCr JOla JOlaNEnNEnJRccJRcc NCrJOlaNEnJRccJRcc JRcc

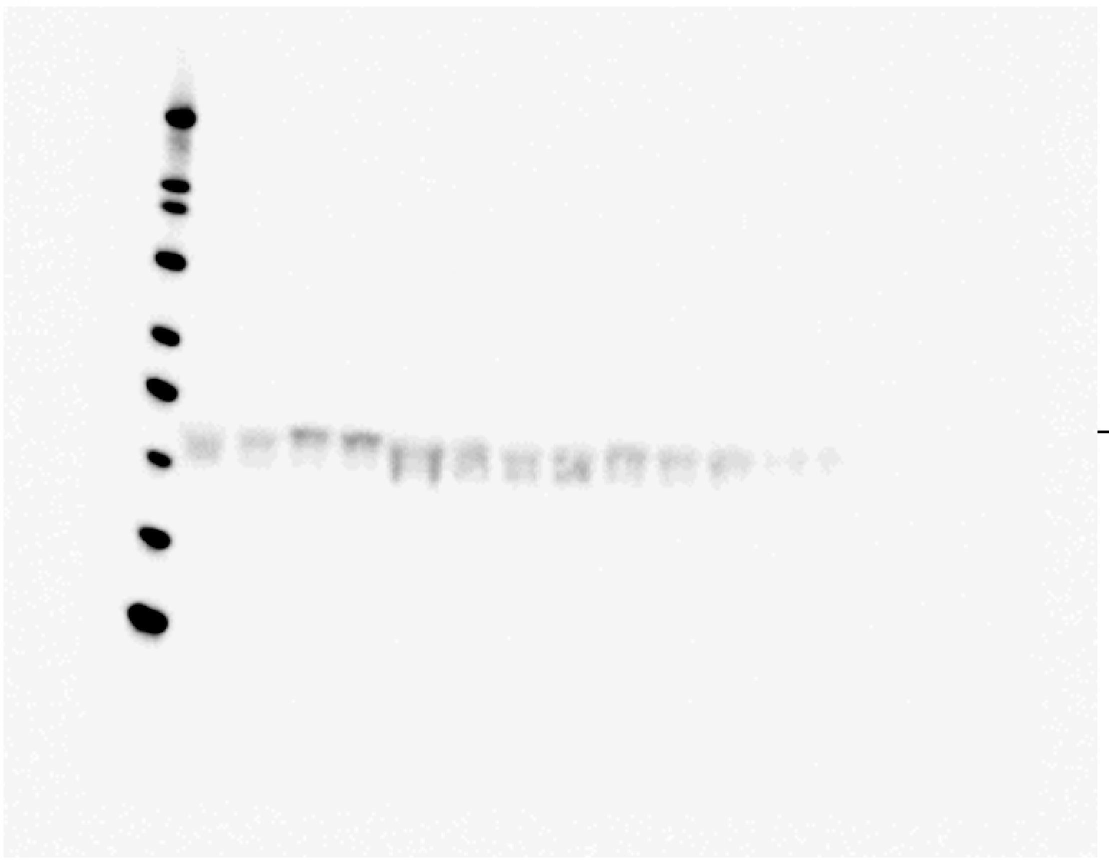

t-GSK3β
46 kDa

**Annotated Groups: MM=Magic Mark XP, JCr =6JCRL (Charles River), NCr =6NCRL, JOla=6JOlaEnv (Envigo), NEn =6NEnv, JRcc=6JRccEnv**

**Method used:** visualised using Enhanced chemiluminescence substrate on iBright™ FL1000 Imaging System camera (Invitrogen™, Fisher Scientific) at 16-bit

# Ponceau for normalisation of p-GSK3β and t-GSK3 β in Fig. 3D

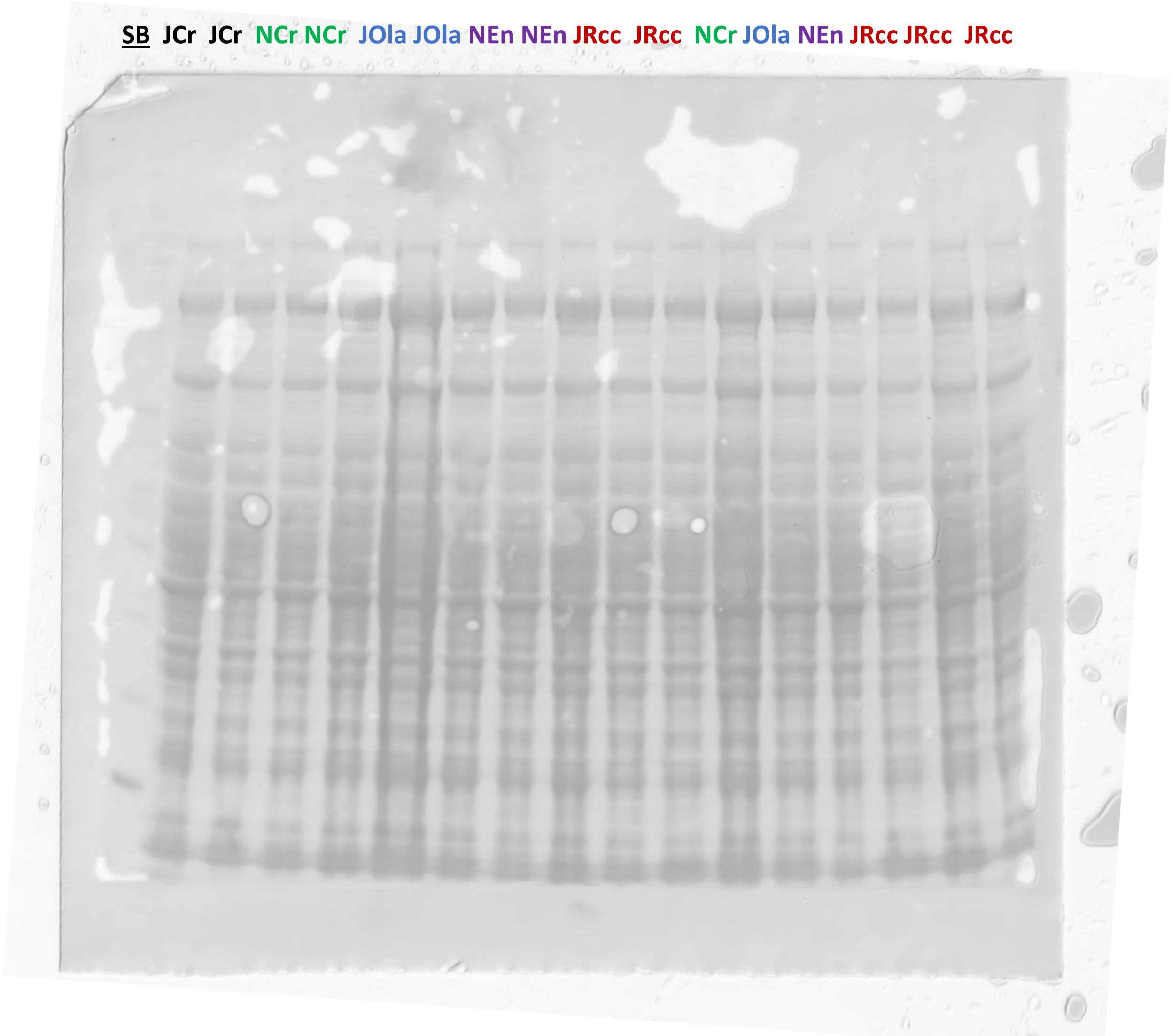


**Annotated Groups:** **SB=Seeblue, JCr =6JCRL (Charles River), NCr =6NCRL, JOla=6JOlaEnv (Envigo), NEn =6NEnv, JRcc=6JRccEnv**

**Method used:** visualised using Enhanced chemiluminescence substrate on iBright™ FL1000 Imaging System camera (Invitrogen™, Fisher Scientific) at 16-bit
