# Supplementary material for: How stra(i)nge are your controls? A comparative analysis of metabolic phenotypes in commonly used C57BL/6 substrains
Source: PLoS One. 2023 Aug 2;18(8):e0289472. doi: 10.1371/journal.pone.0289472 (PMC10395817; doi:10.1371/journal.pone.0289472)
Supplement: S1 Raw images — (PDF) [file pone.0289472.s006.pdf]

Raw blot and total protein stain (Ponceau) images

Manuscript ID: PONE-D-23-11737R1

Female substrain blots (Fig. 3A)

p-AKT for Fig. 3A

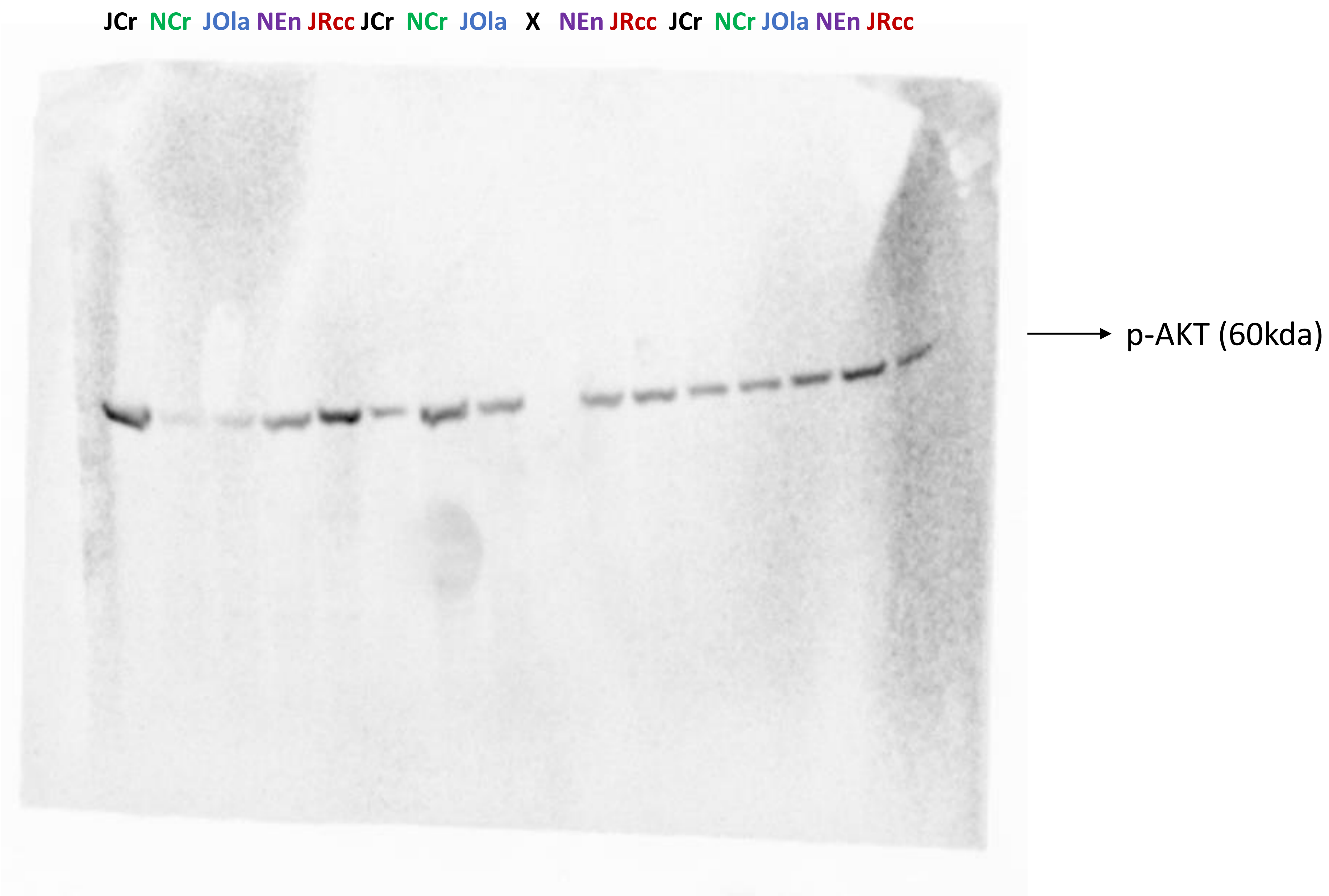

Annotated Groups:
JCr =6JCRL (Charles River)
NCr =6NCRL
JOla=6JOlaEnv (Envigo)
NEn =6NEnv
JRcc=6JRccEnv

Method used:
visualised using Enhanced chemiluminescence substrate on iBright™ FL1000 Imaging System camera (Invitrogen™, Fisher Scientific) at 16-bit

t-AKT for Fig. 3A

JCr NCr JOla NEn JRcc JCr NCr JOla X NEn JRcc JCr NCr JOla NEn JRcc

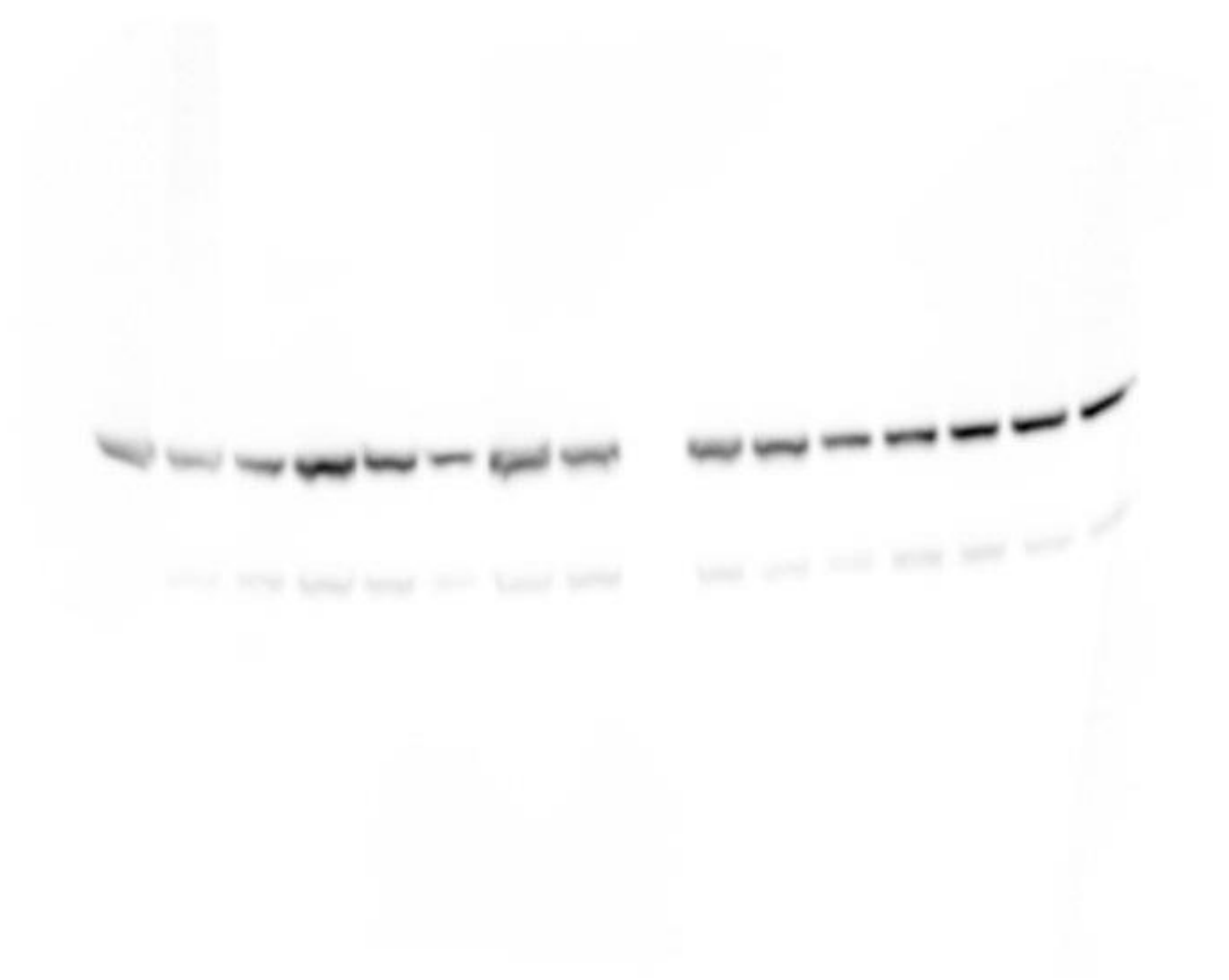

→ t-AKT 60kDa

Annotated Groups:
JCr =6JCRL (Charles River)
NCr =6NCRL
JOla=6JOlaEnv (Envigo)
NEn =6NEnv
JRcc=6JRccEnv

Method used:
visualised using Enhanced chemiluminescencse ubstrate on iBright™ FL1000 Imaging System camera (Invitrogen™, Fisher Scientific) at 16-bit

Ponceau for normalisation of p-AKT and t-AKT in Fig. 3A

<u>SB</u> JCr NCr JOla NEn JRcc JCr NCr JOla X NEn JRcc JCr NCr JOla NEn JRcc

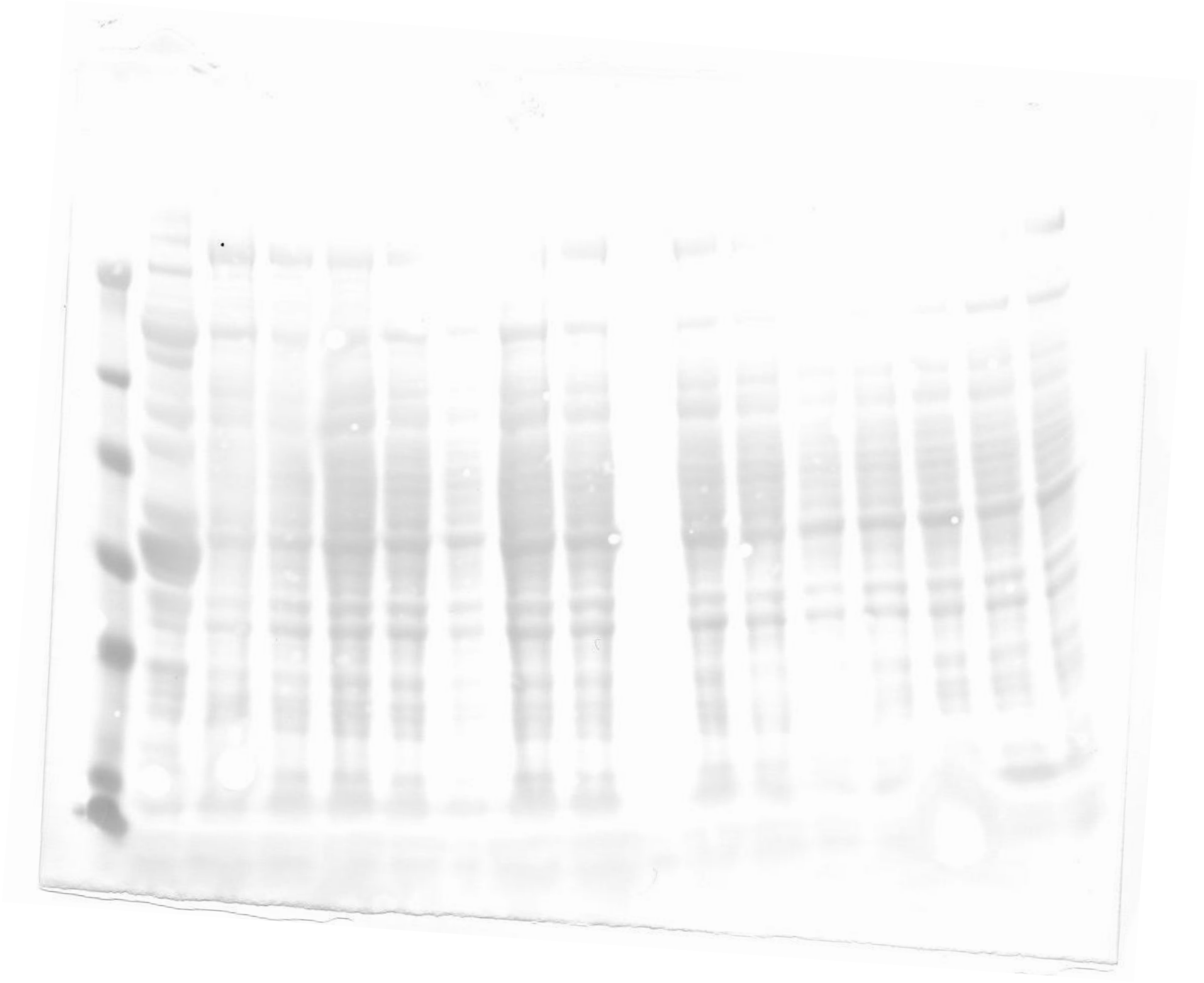

Annotated Groups:

SB=Seeblue
JCr =6JCRL (Charles River)
NCr =6NCRL
JOla=6JOlaEnv (Envigo)
NEn =6NEnv
JRcc=6JRccEnv

Method used:
visualised using Enhanced chemiluminescence substrate on iBright™ FL1000 Imaging System camera (Invitrogen™, Fisher Scientific) at 16-bit

p-rpS6 for Fig. 3A

MM JCr NCr JOla NEn JRcc JCr NCr JOla NEn JRcc JCr NCr JOla NEn JRcc

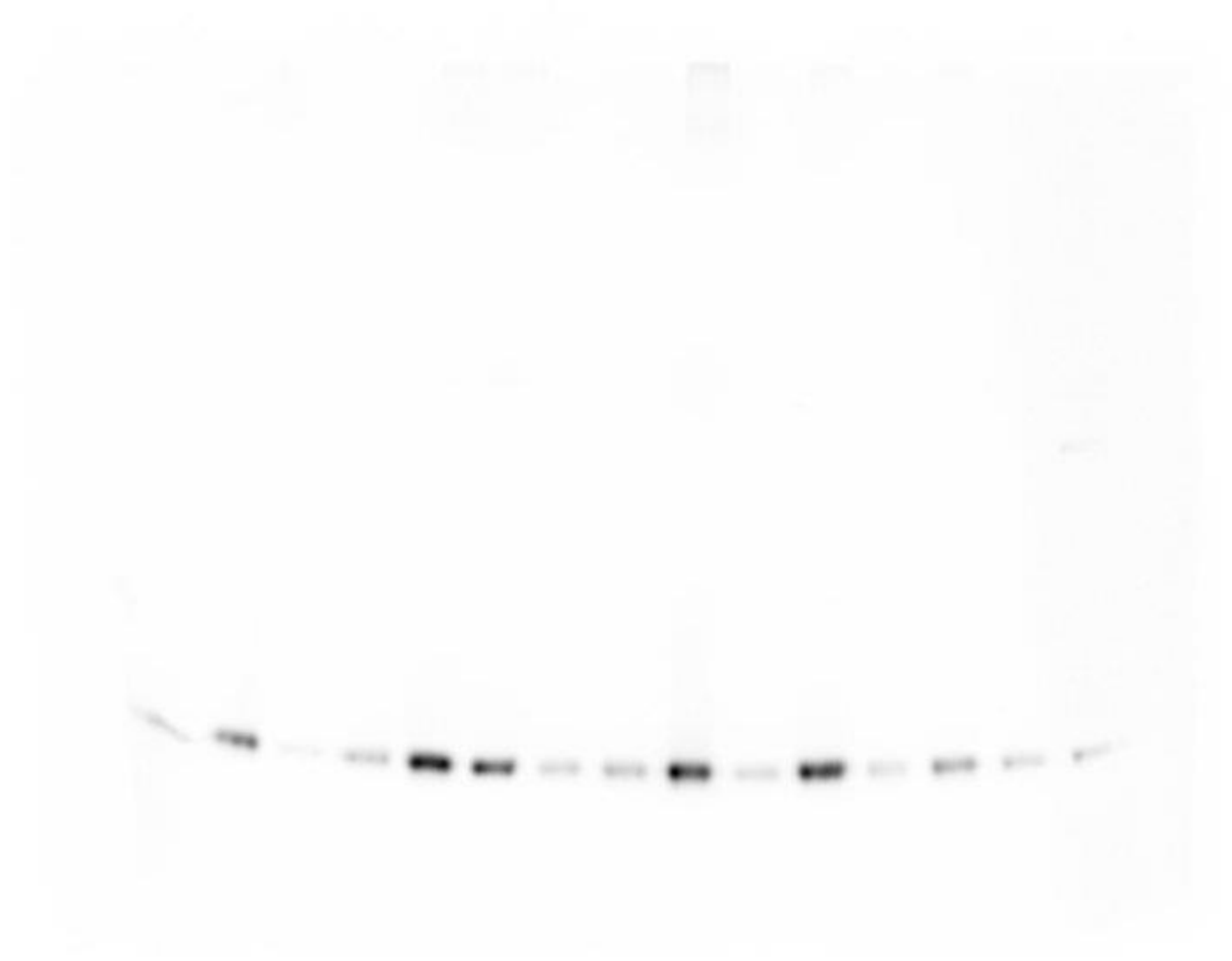

p-rpS6
32kDa

Annotated Groups:
MM=Magic Mark XP
JCr =6JCRL (Charles River)
NCr =6NCRL
JOla=6JOlaEnv (Envigo)
NEn =6NEnv
JRcc=6JRccEnv

Method used:
visualised using Enhanced chemiluminescence substrate on iBright™ FL1000 Imaging System camera (Invitrogen™, Fisher Scientific) at 16-bit

t-rpS6 for Fig. 3A

MM JCr NCr JOla NEn JRcc JCr NCr JOla NEn JRcc JCr NCr Jola NEn JRcc

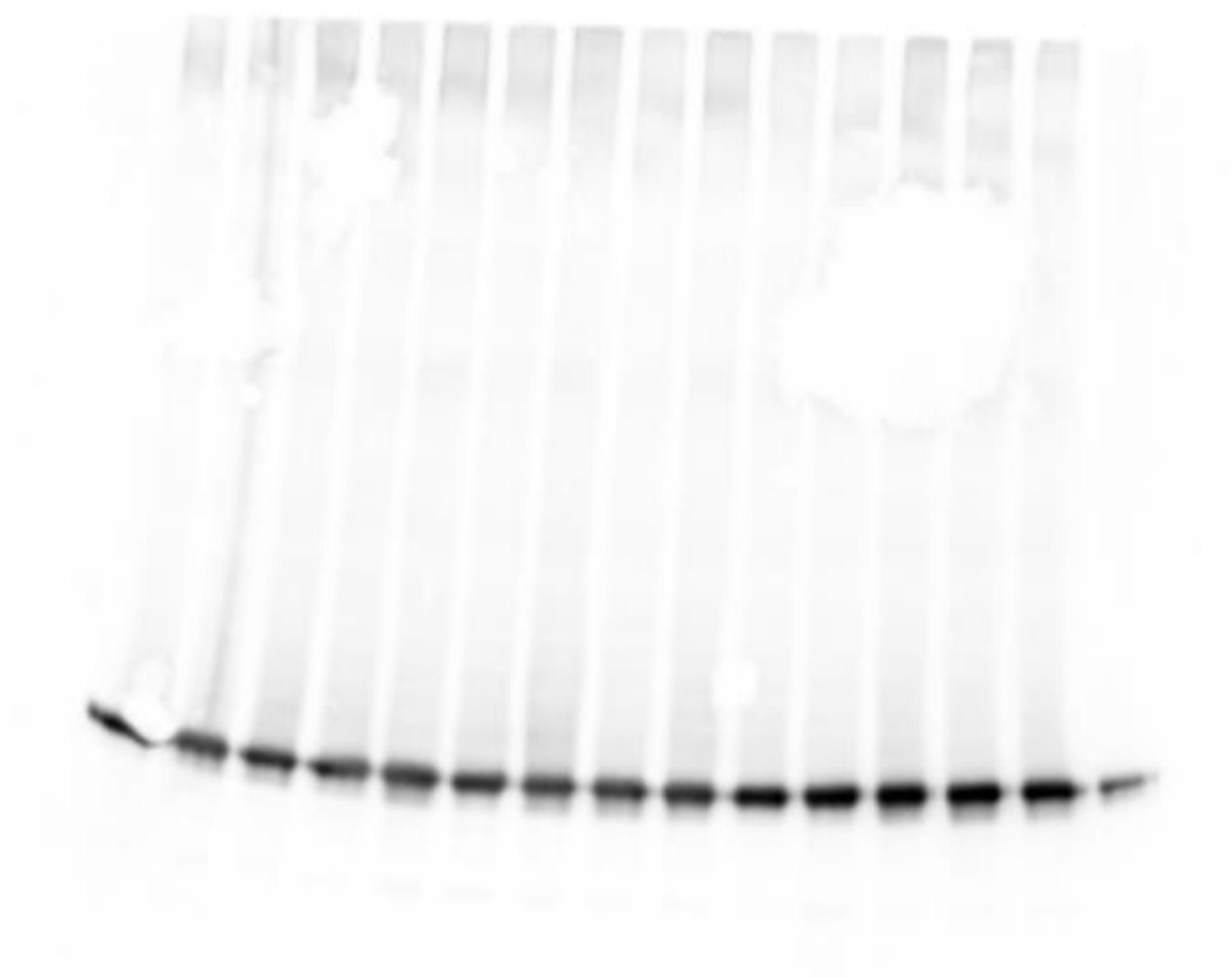

t-rpS6
32 kDa

Annotated Groups:
MM=Magic Mark XP
JCr =6JCRL (Charles River)
NCr =6NCRL
JOla=6JOlaEnv (Envigo)
NEn =6NEnv
JRcc=6JRccEnv

Method used:
visualised using Enhanced chemiluminescencs substrate on iBright™ FL1000 Imaging System camera (Invitrogen™, Fisher Scientific) at 16-bit

p-GSK3β for Fig. 3A

MM JCr NCr JOla NEn JRcc JCr NCr JOla NEn JRcc JCr NCr JOla NEn JRcc

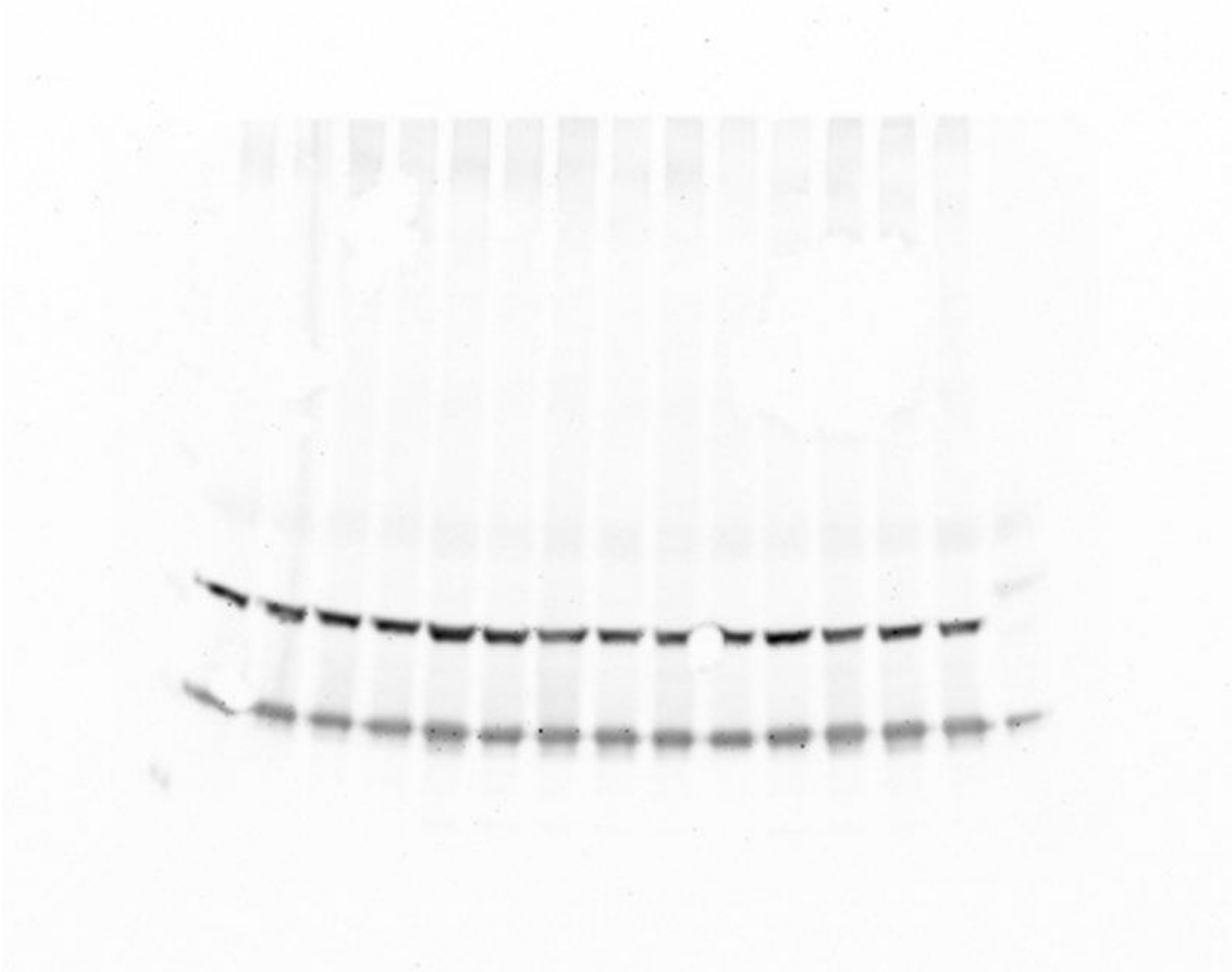

p-GSK3β
46 kDa

Annotated Groups:
MM=Magic Mark XP
JCr =6JCRL (Charles River)
NCr =6NCRL
JOla=6JOlaEnv (Envigo)
NEn =6NEnv
JRcc=6JRccEnv

Method used:
visualised using Enhanced chemiluminescencssubstrate on iBright™ FL1000 Imaging System camera (Invitrogen™, Fisher Scientific) at 16-bit

t-GSK3β for Fig. 3A

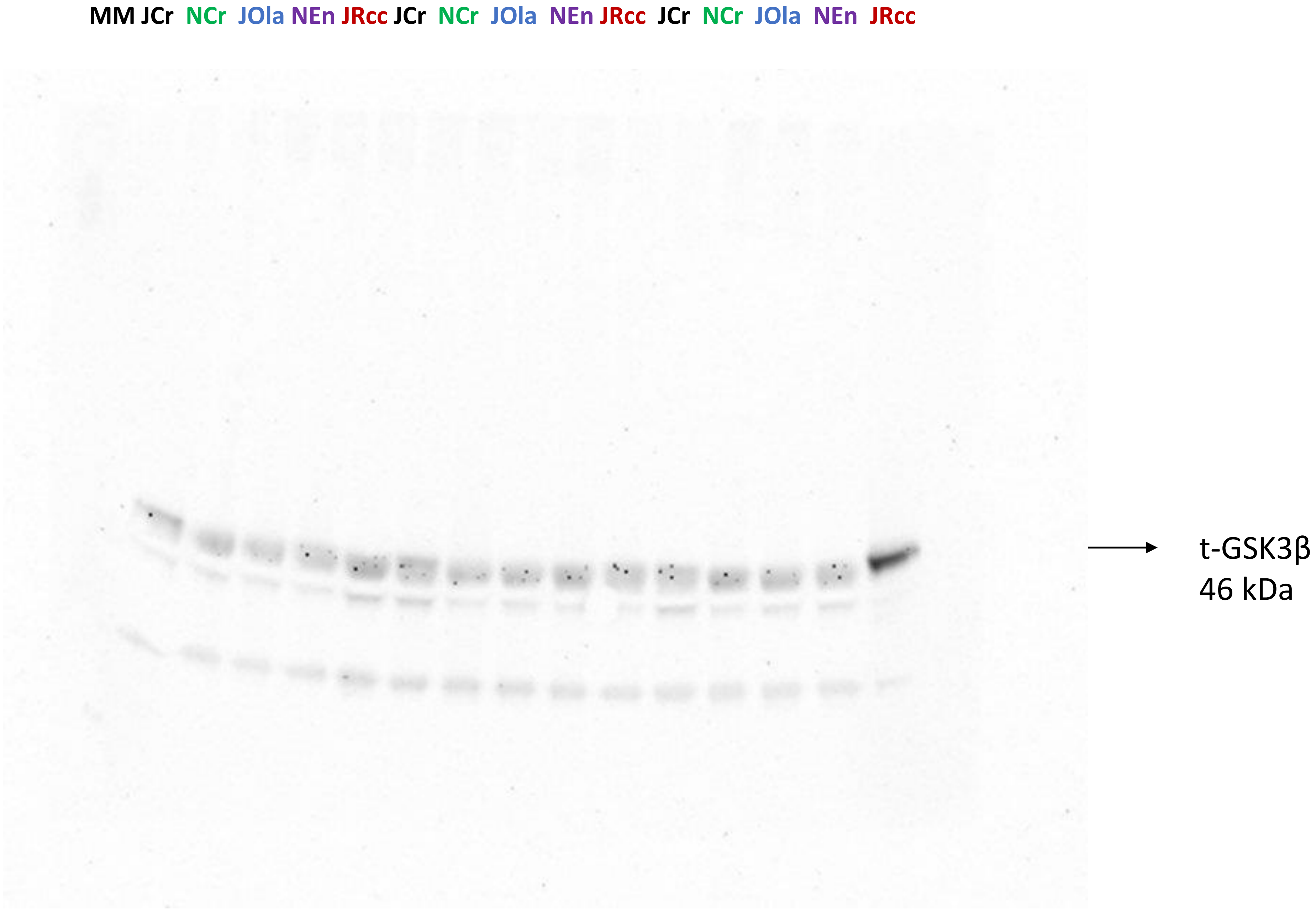

Annotated Groups:
MM=Magic Mark XP
JCr =6JCRL (Charles River)
NCr =6NCRL
JOla=6JOlaEnv (Envigo)
NEn =6NEnv
JRcc=6JRccEnv

Method used:
visualised using Enhanced chemiluminescencssubstrate on iBright™ FL1000 Imaging System camera (Invitrogen™, Fisher Scientific) at 16-bit

Ponceau for normalisation of AKT and GSK3B (both p- and t-)in Fig. 3A

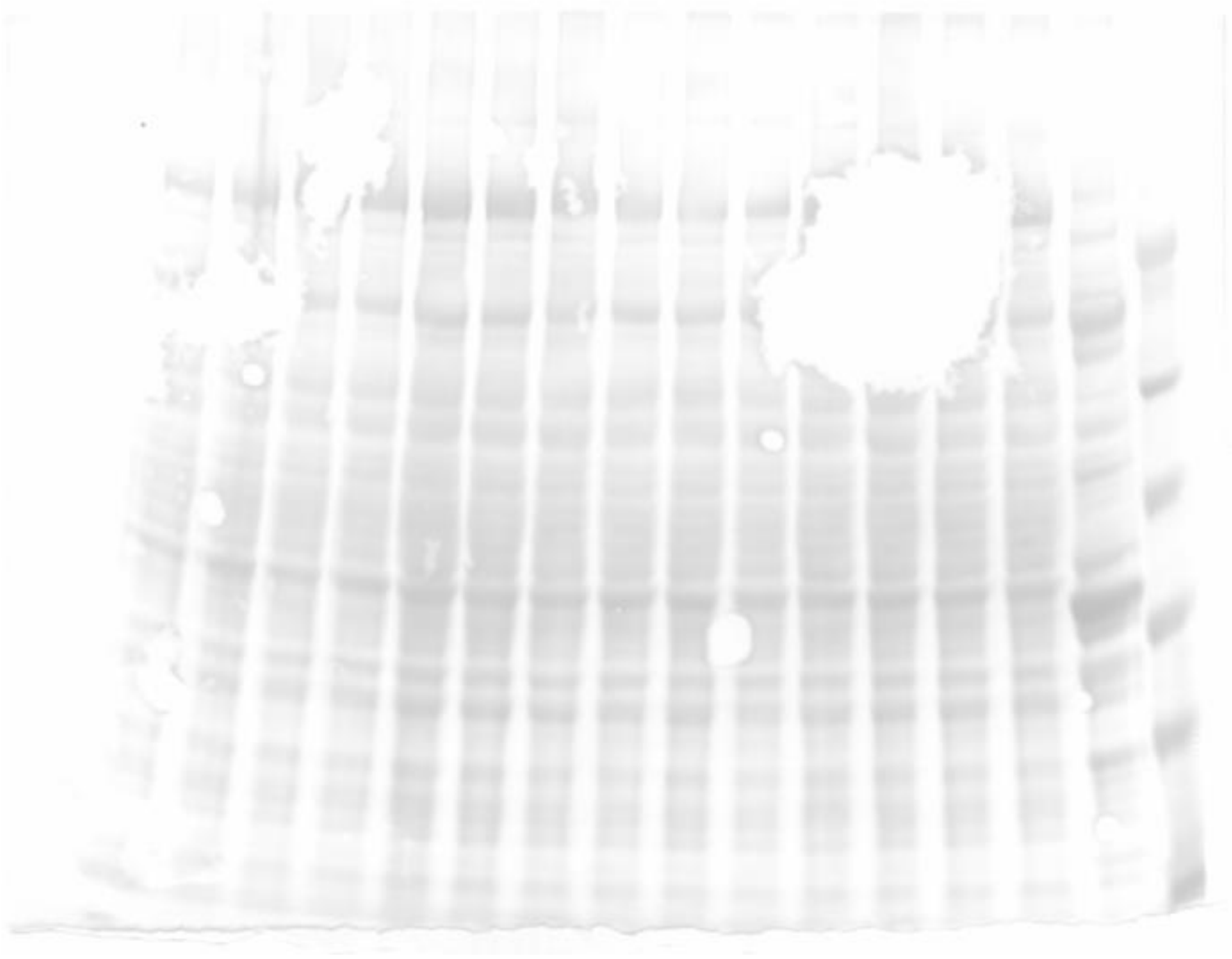

Annotated Groups:
JCr =6JCRL (Charles River)
NCr =6NCRL
JOla=6JOlaEnv (Envigo)
NEn =6NEnv
JRcc=6JRccEnv
SB = Seeblue

Method used:
visualised using Enhanced chemiluminescence substrate on iBright™ FL1000 Imaging System camera (Invitrogen™, Fisher Scientific) at 16-bit

Male substrain blots (Fig. 3D)

p-AKT for Fig. 3D

MM JCr JCr NCr NCr JOla NEnNEn JRcc JRcc JCr NCrJOlaNEnJRcc JRcc

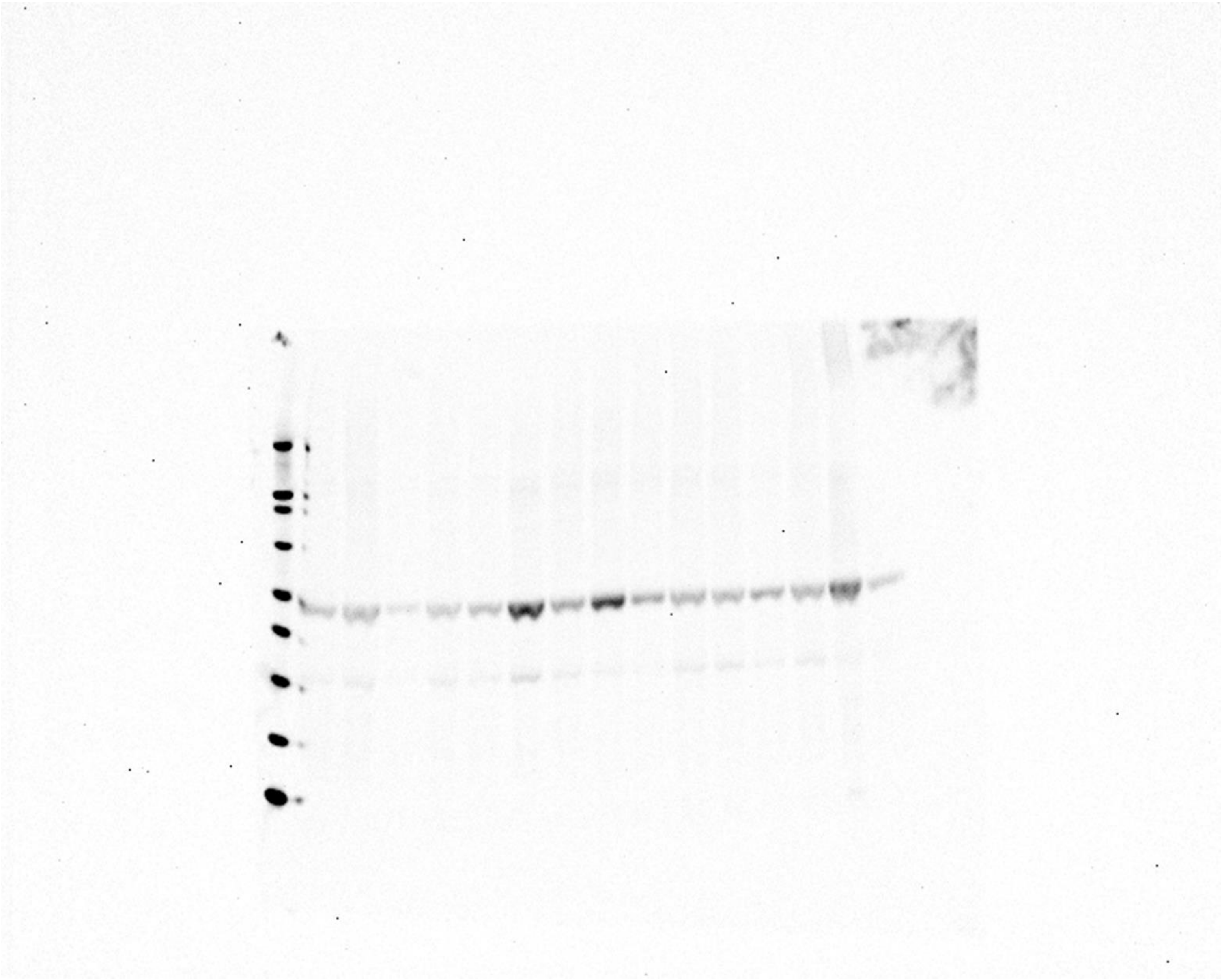

p-AKT
60 kDa

Annotated Groups:
MM=Magic Mark XP
JCr =6JCRL (Charles River)
NCr =6NCRL
JOla=6JOlaEnv (Envigo)
NEn =6NEnv
JRcc=6JRccEnv

Method used:
visualised using Enhanced chemilumiscence substrate on iBright™ FL1000 Imaging System camera (Invitrogen™, Fisher Scientific) at 16-bit

t-AKT for Fig. 3D

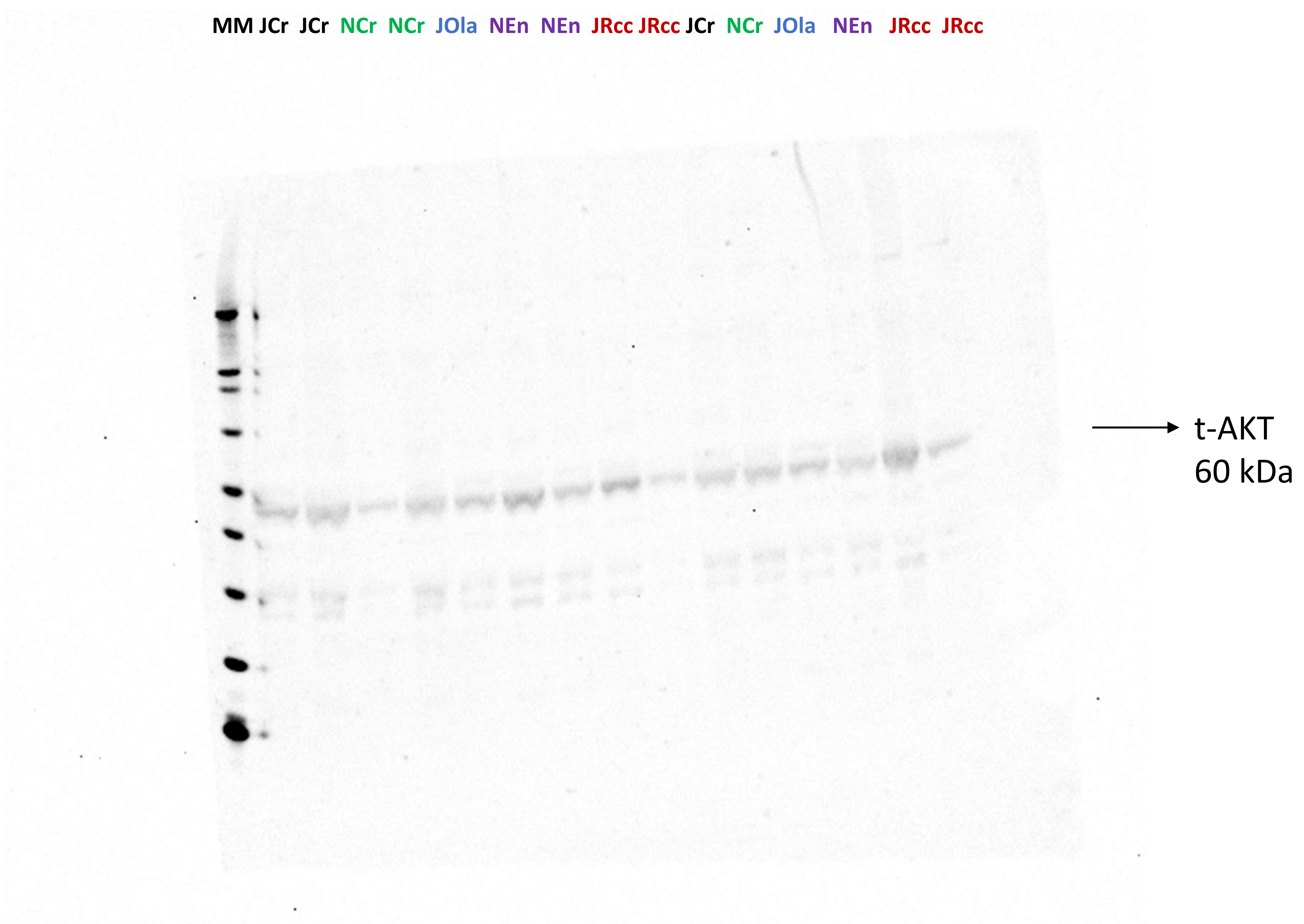

Annotated Groups:
MM=Magic Mark XP
JCr =6JCRL (Charles River)
NCr =6NCRL
JOla=6JOlaEnv (Envigo)
NEn =6NEnv
JRcc=6JRccEnv

Method used:
visualised using Enhanced chemiluminescence substrate on iBright™ FL1000 Imaging System camera (Invitrogen™, Fisher Scientific) at 16-bit

Ponceau for normalisation of p-AKT and t-AKT in Fig. 3D

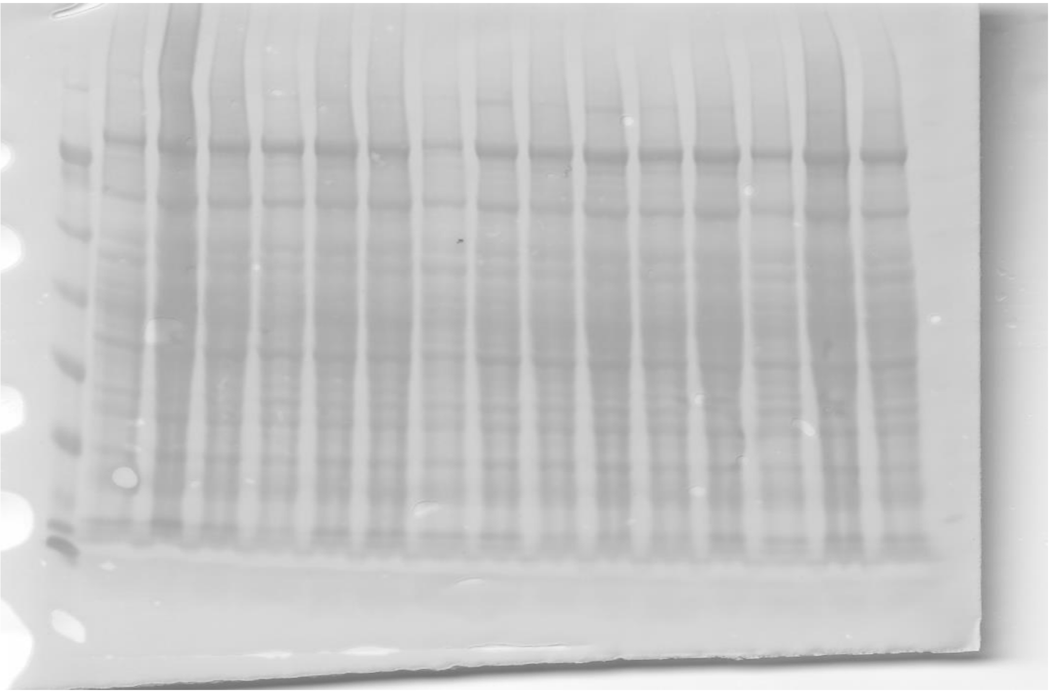

Annotated Groups:
MM=Magic Mark XP
JCr =6JCRL (Charles River)
NCr =6NCRL
JOla=6JOlaEnv (Envigo)
NEn =6NEnv
JRcc=6JRccEnv

Method used:
visualised using Enhanced chemilumiscence substrate on iBright™ FL1000 Imaging System camera (Invitrogen™, Fisher Scientific) at 16-bit

p-rpS6 for Fig. 3D

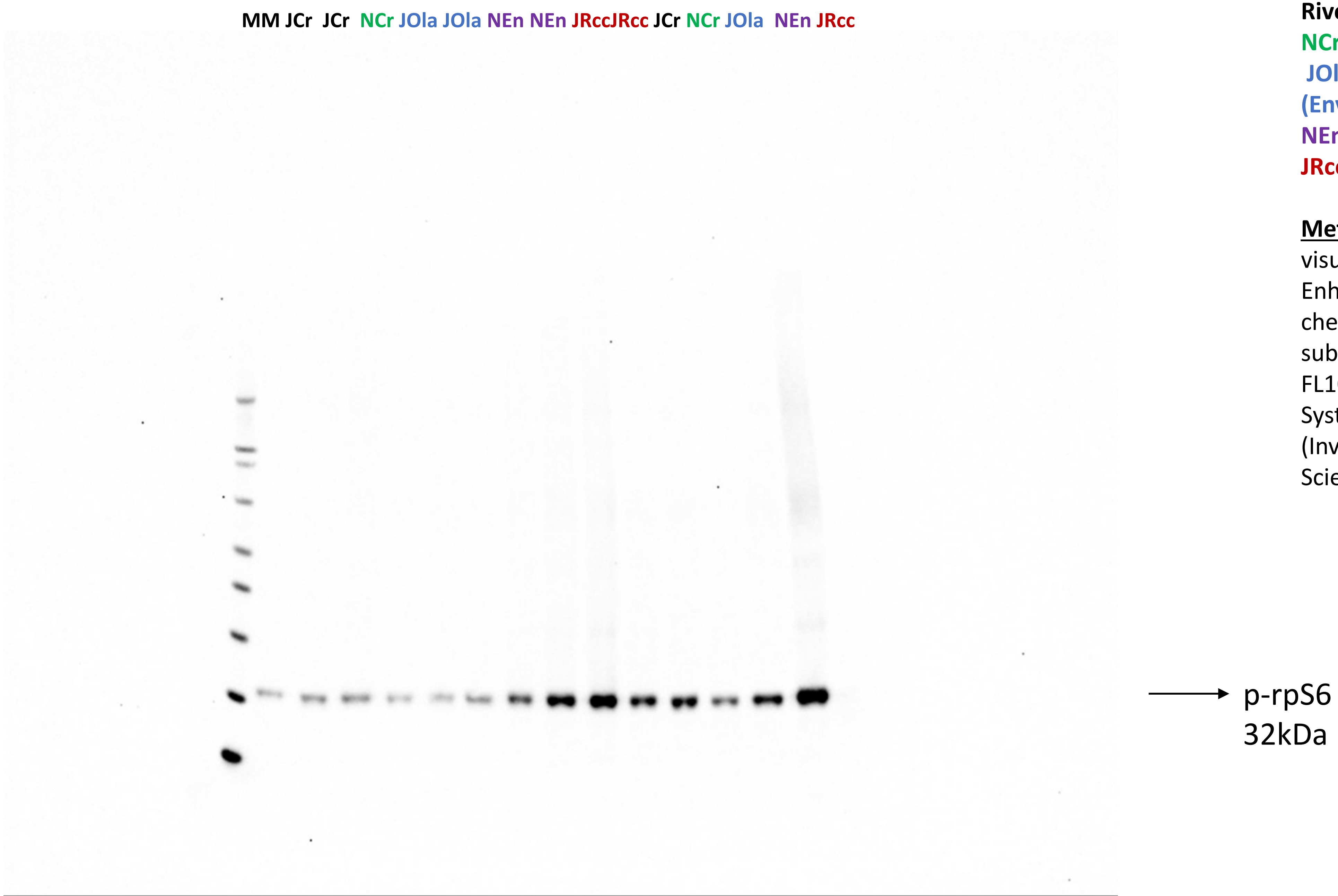

Annotated Groups:
MM=Magic Mark XP
JCr =6JCRL (Charles River)
NCr =6NCRL
JOla=6JOlaEnv (Envigo)
NEn =6NEnv
JRcc=6JRccEnv

Method used:
visualised using Enhanced chemiluminescence substrate on iBright™ FL1000 Imaging System camera (Invitrogen™, Fisher Scientific) at 16-bit

t-rpS6 for Fig. 3D

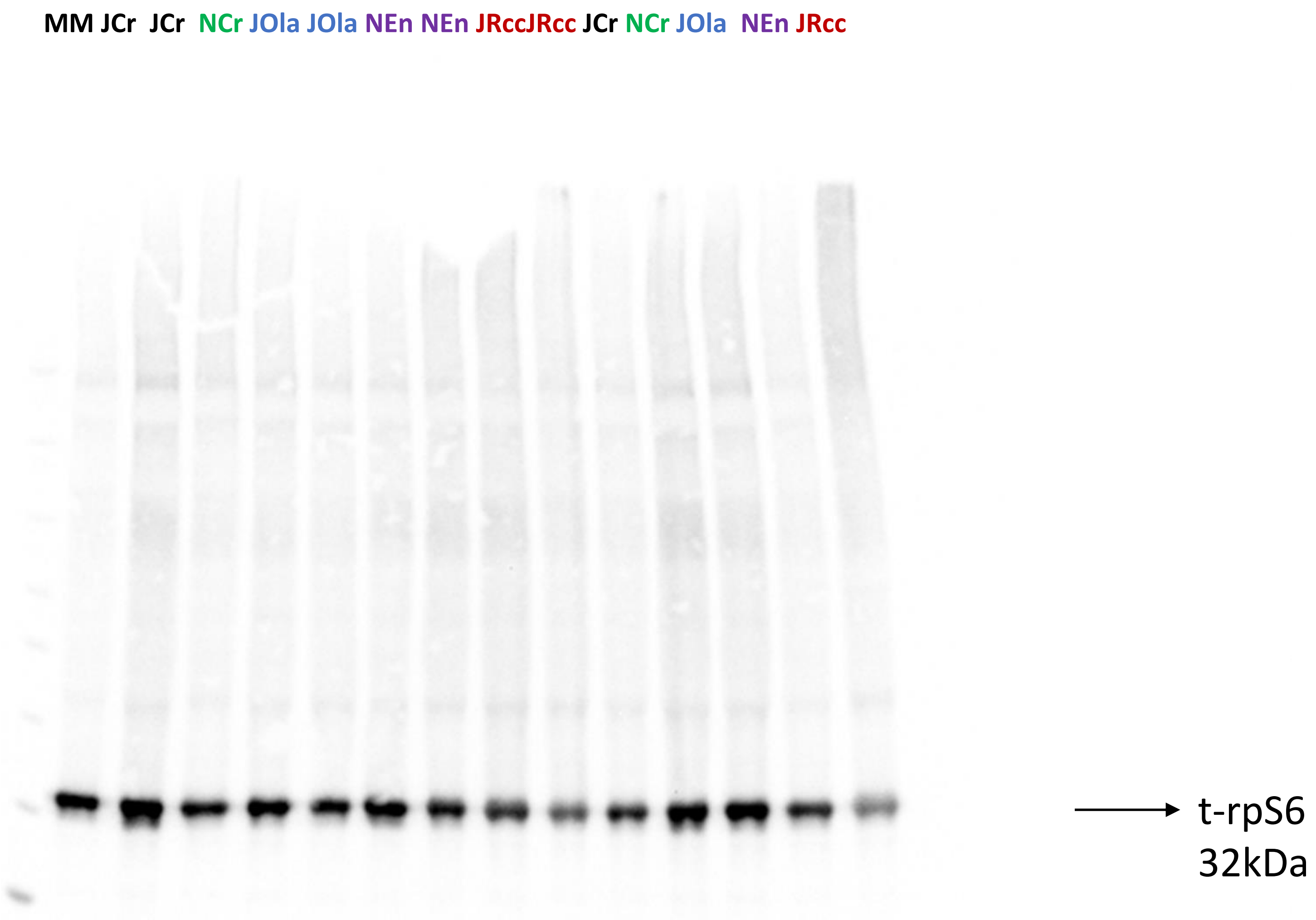

Annotated Groups:
MM=Magic Mark XP
JCr =6JCRL (Charles River)
NCr =6NCRL
JOla=6JOlaEnv (Envigo)
NEn =6NEnv
JRcc=6JRccEnv

Method used:
visualised using Enhanced chemiluminescence substrate on iBright™ FL1000 Imaging System camera (Invitrogen™, Fisher Scientific) at 16-bit

Ponceau for normalisation of p-rpS6 and t-rpS6 in Fig. 3D

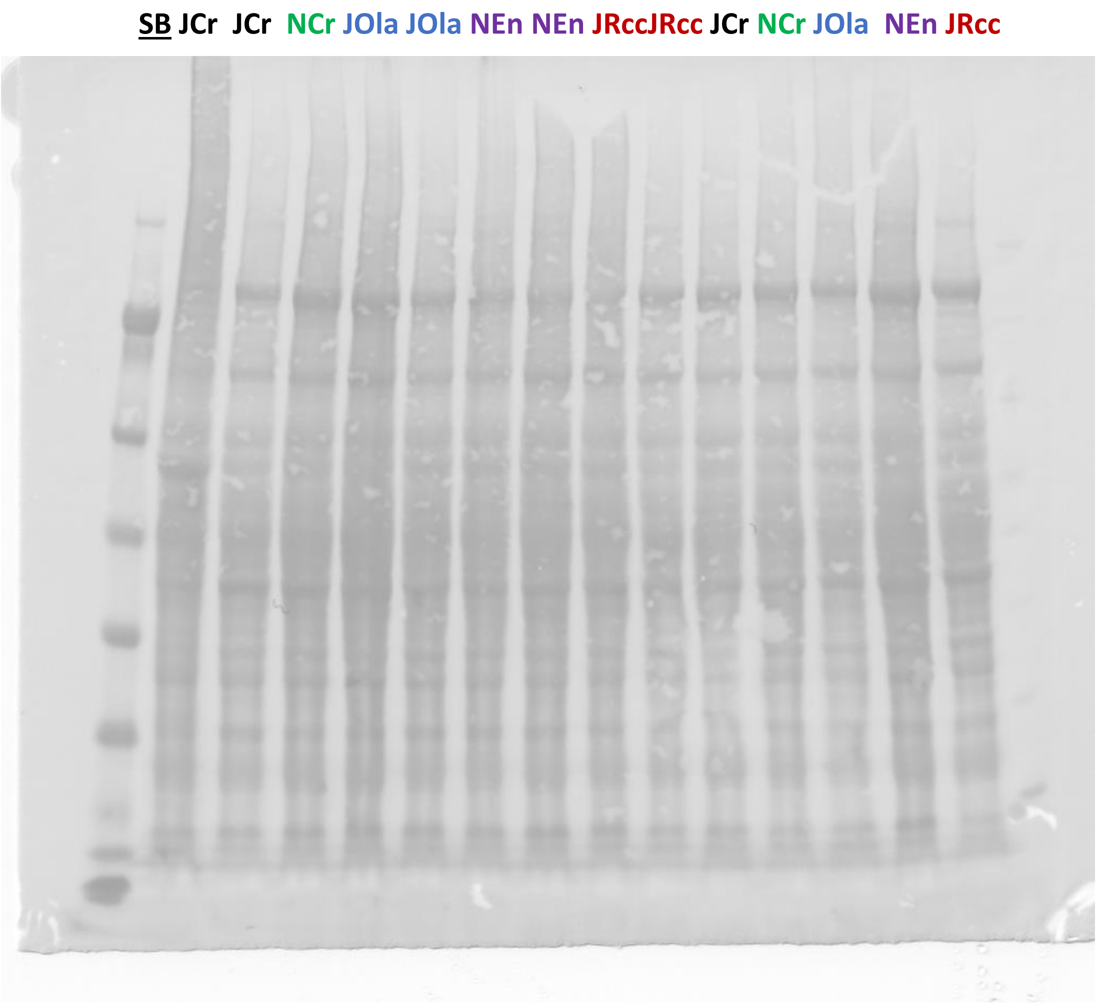

Annotated Groups:
MM=Magic Mark XP
SB = Seeblue
JCr =6JCRL (Charles River)
NCr =6NCRL
JOla=6JOlaEnv (Envigo)
NEn =6NEnv
JRcc=6JRccEnv

Method used:
visualised using Enhanced chemiluminescence substrate on iBright™ FL1000 Imaging System camera (Invitrogen™, Fisher Scientific) at 16-bit

p-GSK3β for Fig. 3D

<u>Original blot photo from camera</u>

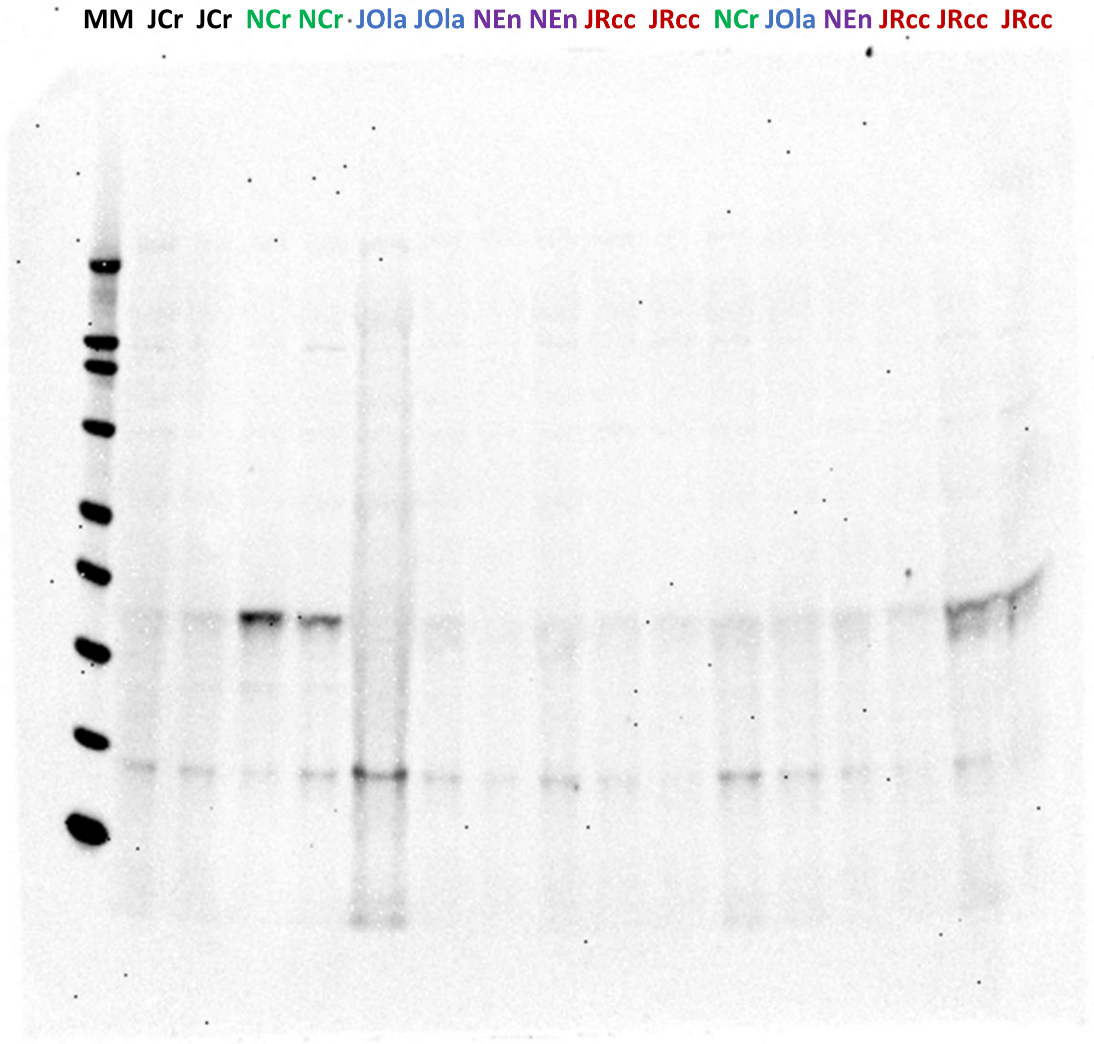

<u>Contrast ramped up on Image J to analyze, matching blot photo in Fig 3D</u>

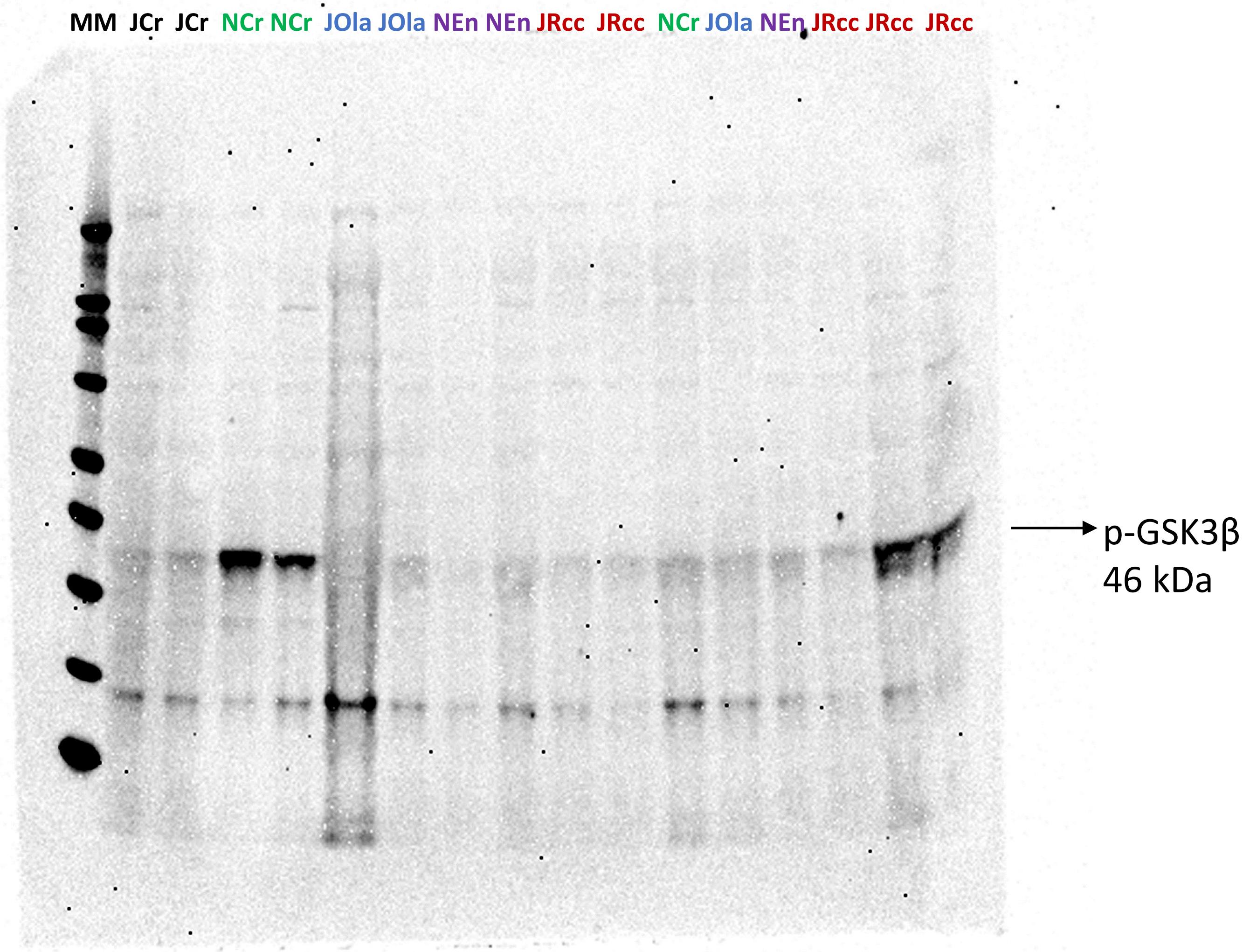

Annotated Groups: **MM=Magic Mark XP, JCr =6JCRL (Charles River), NCr =6NCRL, JOla=6JOlaEnv (Envigo), NEn =6NEnv, JRcc=6JRccEnv**

<u>Method used:</u> visualised using Enhanced chemiluminescence substrate on iBright™ FL1000 Imaging System camera (Invitrogen™, Fisher Scientific) at 16-bit

t-GSK3β for Fig. 3D

Original blot photo from camera

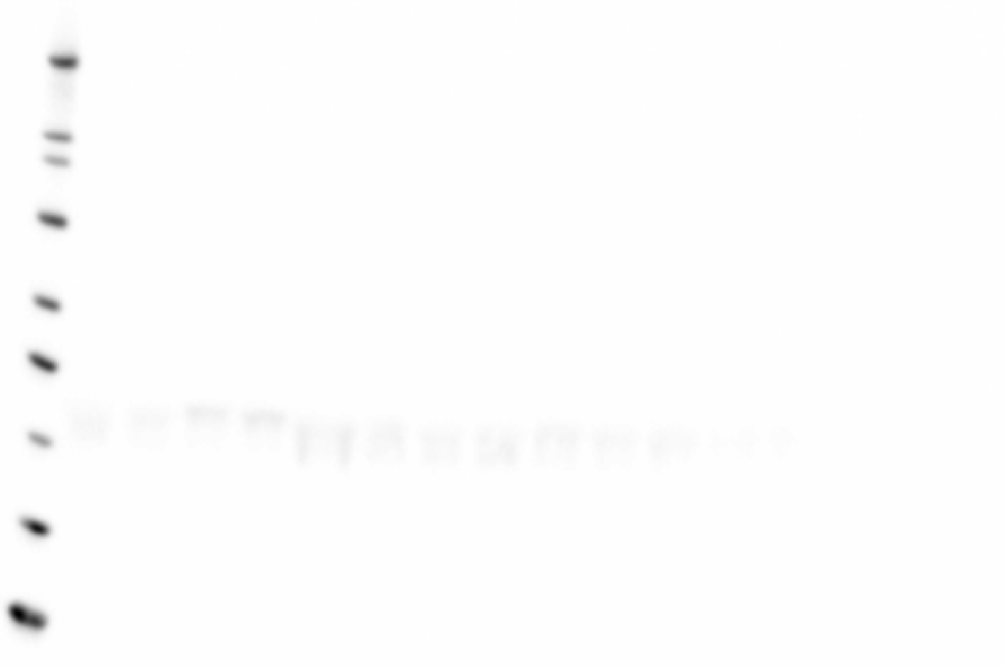

Contrast ramped up on Image J to analyze, matching blot photo in Fig 3D

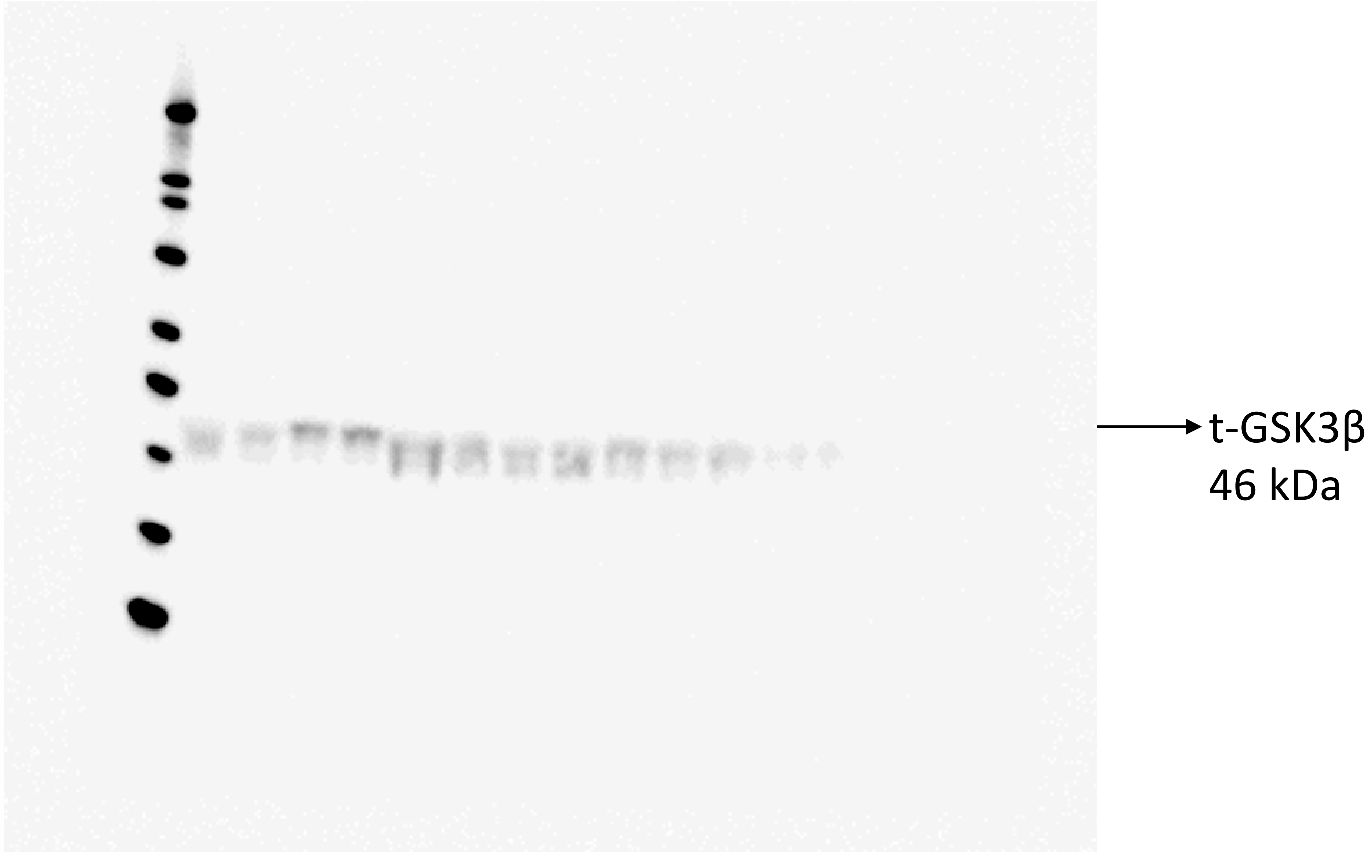

Annotated Groups: MM=Magic Mark XP, JCr =6JCRL (Charles River), NCr =6NCRL, JOla=6JOlaEnv (Envigo), NEn =6NEnv, JRcc=6JRccEnv

Method used: visualised using Enhanced chemiluminescence substrate on iBright™ FL1000 Imaging System camera (Invitrogen™, Fisher Scientific) at 16-bit

Ponceau for normalisation of p-GSK3β and t-GSK3 β in Fig. 3D

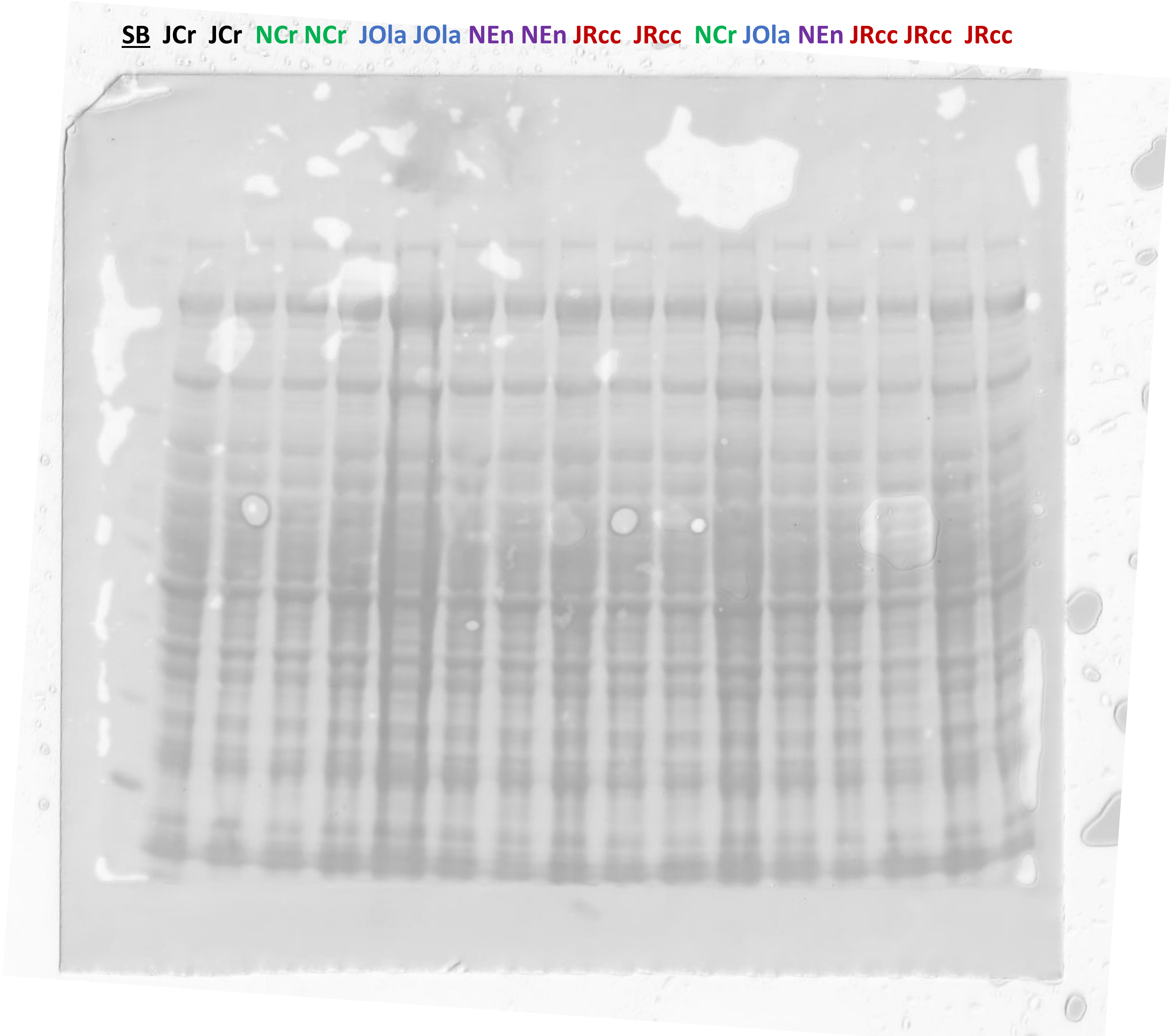

Annotated Groups: **SB=Seeblue, JCr =6JCRL (Charles River), NCr =6NCRL, JOla=6JOlaEnv (Envigo), NEn =6NEnv, JRcc=6JRccEnv**

Method used: visualised using Enhanced chemiluminescence substrate on iBright™ FL1000 Imaging System camera (Invitrogen™, Fisher Scientific) at 16-bit
