# Supplementary material for: How stra(i)nge are your controls? A comparative analysis of metabolic phenotypes in commonly used C57BL/6 substrains
Source: PLoS One. 2023 Aug 2;18(8):e0289472. doi: 10.1371/journal.pone.0289472 (PMC10395817; doi:10.1371/journal.pone.0289472)
Supplement: Attachment — Submitted filename: Response to the reviewers.docx [file pone.0289472.s009.docx]

Response to the reviewers

Manuscript ID: PONE-D-23-11737R1

‘How stra(i)nge are your controls? A comparative analysis of metabolic phenotypes in commonly used C57 substrains’

Dear Prof. Pizzo,

Thank you very much for considering our manuscript for publication in your journal, and for the encouraging news on our submission.

We are grateful to the reviewers for their valuable comments and appreciations. All comments have been carefully considered, and the manuscript has been amended according to the suggestions made. Please find a point-by-point overview attached.

The minimum underlying data set which includes raw Excel data files for the Glucose Tolerance Test, serum insulin ELISA, raw images of both total protein Stain Ponceau and raw images of the blots have now been uploaded as Supporting Information files to the manuscript.

As requested by the reviewers, two new references have been added to the reference list (PMID: 26299453, doi: 10.1089/ars.2021.0111). The reference list has also been updated as requested.

We hope the manuscript is now suitable for publication, but please do not hesitate to contact us if there are any further questions.

We would like to thank you for the speedy processing of our submission and trust that PLOS One readers will find our contribution interesting.

Sincerely,

Annesha Sil and Bettina Platt

Reviewer #1:

**We are very pleased that the reviewer considers this to be a well-conducted and strong study and one which addresses an important aspect of animal research.**

Replies to specific points follows:

1-Basal glycemia and insulin levels could be presented in Fig 1 with BW data while GTT and AUC data presented as Fig 2. It sounds more logical to this reviewer at least.

**Reply: We thank the reviewer for this comment. We would, however, prefer to maintain the figures as they currently are. For us, maintaining Fig.1 with body weight and glucose tolerance and AUC seems more logical as these were evaluated earlier in the experimental timeline, whereas serum insulin was evaluated post-mortem. We do understand that basal glucose was also evaluated during the glucose tolerance test however we did not want to increase the number of figures for the manuscript to keep it as succinct as possible and hence this has been combined with the fasted insulin levels in Fig. 2.**

2-There are evidence in the literature that microbiota could be involved in metabolic differences between mouse strains and substrains. See for instance PMID: 26299453. This aspect should be discussed also as a potential contributor to the differences observed in the current study.

**Reply: Indeed, the reviewer raises an important point. Accordingly, an additional line has been added in the discussion from line 363-365 with the reference the reviewer has mentioned as follows: ‘Additionally, while not investigated in the current study, differences in gut microbiota of individual strains has been implicated in metabolic alterations between substrains and should be considered for future studies [38].’**

Reviewer #2:

**We thank the reviewer for their valuable suggestions.**

Replies to specific points follows:

- Manuscript title: “C57BL/6 substrains” should be more appropriate than “C57 substrains”.

**Reply: This has now been amended.**

- Manuscript text: please replace “sub-strains” with “substrains”.

**Reply: This has now been altered as requested.**

- Define the Nnt mutation as Nnt^C57BL/6J when it first appears (on line 29). See [https://www.informatics.jax.org/allele/MGI:3626282](https://eur03.safelinks.protection.outlook.com/?url=https%3A%2F%2Fwww.informatics.jax.org%2Fallele%2FMGI%3A3626282&data=05%7C01%7Cannesha.sil%40abdn.ac.uk%7Cd4ba99b166fd4f937a4e08db70a08c28%7C8c2b19ad5f9c49d490773ec3cfc52b3f%7C0%7C0%7C638227607073815127%7CUnknown%7CTWFpbGZsb3d8eyJWIjoiMC4wLjAwMDAiLCJQIjoiV2luMzIiLCJBTiI6Ik1haWwiLCJXVCI6Mn0%3D%7C3000%7C%7C%7C&sdata=mkVVB%2BkYatzhSHRnRL58G2c6caNkAYjkdePKtarXsGk%3D&reserved=0)

**Reply: This has now been changed.**

- Line 60: Remove “(NAD)”

**Reply: This has now been changed.**

- Line 61: Please remove “, required for ATP synthesis”.
- Line 61: NADPH instead of NAPDH.
- Line 61: “NADPH is required for biosynthesis and redox homeostasis” instead of “NAPDH detoxifies reactive oxygen species (ROS) in mitochondria, essential for H2O2 disposal and redox homeostasis”.

**Reply: This line has been changed as requested.**

- Results section: Remove figure captions (Figures 1-4) from the text.

**Reply: We apologise, however this cannot be altered as we were just following the journals submission guidelines which request the caption to be placed right after the paragraph in which it first appears**

- I suggest authors cite a critical review on mouse background strain and the history of the Nnt mutation in C57BL/6J:
Diabetes. 2016 Jan;65(1):25-33. doi: 10.2337/db15-0982.

**Reply: This critical review was already in the reference list (Reference no. 2, line 46). We apologise if this was unclear in any form.**

- I suggest that the authors cite a critical review on the effects of the Nnt mutation on mouse obesity and several other parameters:
Antioxid Redox Signal. 2022 May;36(13-15):864-884. doi: 10.1089/ars.2021.0111.

**Reply: We thank the reviewer for this suggestion. This has now been added to line 64 as an additional reference number [11].**
